# Open Knee: Open Source Modeling & Simulation to Enable Scientific Discovery and Clinical Care in Knee Biomechanics

**DOI:** 10.1055/s-0035-1564600

**Published:** 2015-10-07

**Authors:** Ahmet Erdemir

**Affiliations:** 1 Computational Biomodeling (CoBi) Core, Lerner Research Institute, Cleveland Clinic, Cleveland, OH, USA; 2 Department of Biomedical Engineering, Cleveland Clinic, Cleveland, OH, USA

**Keywords:** knee, biomechanics, clinical biomechanics, tibiofemoral joint, cartilage, meniscus, ligament, passive flexion, laxity, anterior cruciate ligament, meniscectomy, joint movement, tissue mechanics, computational model, finite element analysis, open source, free access, public dissemination

## Abstract

Virtual representations of the knee joint can provide clinicians, scientists, and engineers the tools to explore mechanical function of the knee and its tissue structures in health and disease. Modeling and simulation approaches such as finite element analysis also provide the possibility to understand the influence of surgical procedures and implants on joint stresses and tissue deformations. A large number of knee joint models are described in the biomechanics literature. However, freely accessible, customizable, and easy-to-use models are scarce. Availability of such models can accelerate clinical translation of simulations, where labor intensive reproduction of model development steps can be avoided. The interested parties can immediately utilize readily available models for scientific discovery and for clinical care. Motivated by this gap, this study aims to describe an open source and freely available finite element representation of the tibiofemoral joint, namely Open Knee, which includes detailed anatomical representation of the joint's major tissue structures, their nonlinear mechanical properties and interactions. Three use cases illustrate customization potential of the model, its predictive capacity, and its scientific and clinical utility: prediction of joint movements during passive flexion, examining the role of meniscectomy on contact mechanics and joint movements, and understanding anterior cruciate ligament mechanics. A summary of scientific and clinically directed studies conducted by other investigators are also provided. The utilization of this open source model by groups other than its developers emphasizes the premise of model sharing as an accelerator of simulation-based medicine. Finally, the imminent need to develop next generation knee models are noted. These are anticipated to incorporate individualized anatomy and tissue properties supported by specimen-specific joint mechanics data for evaluation, all acquired in vitro from varying age groups and pathological states.

## Background & Motivation

Computational modeling and simulation has become an integral component of knowledge discovery in biomedical sciences. Virtual representations of the body have also supported accurate and effective delivery of healthcare. As a result, the premise of simulation-based approaches has been promoted by government agencies in the Unites States to accelerate scientific innovations^1^ and to deliver training, and specifically from a healthcare stand-point, to streamline the design, evaluation, and regulation of medical interventions^2^.

Musculoskeletal biomechanics community recognized and has exploited the power of modeling and simulation. At one end of the modeling and simulation spectrum, musculoskeletal movement simulations have been common. In this modeling modality, rigid body representations of the extremities are combined with simplified mechanical representations of joints and muscles to provide an in-depth understanding of human movement and its control^3,4^. Recent advancements in modeling strategies allowed incorporation of more elaborate representations of the knee joint for multi-body dynamics based simulations of the musculoskeletal system^5–7^. At the other end of the spectrum, finite element analysis has been a common modeling and simulation strategy^4,8^. With this tool, anatomical realism of the joint and its tissue structures can be represented through the discretization of tissue volumes into a mesh – a collection of simple geometric shapes, aka elements, connected to each other by nodes. After assigning mechanical properties to tissues, defining interactions in between, e.g., contact, and prescribing loading and boundary conditions, simulations can be conducted to predict not only tissue stresses and strains but also the emerging joint mechanical behavior.

For the knee joint, finite element analysis found many uses to understand the individual role of tissue components on knee mechanics^9–11^. From a clinical perspective, the simulations have been utilized to explore injury mechanisms^12,13^, to evaluate mechanical impact of pathological conditions such as osteoarthritis^14^, to assess the performance and secondary effects of surgical interventions^15–17^, and to design and evaluate implants^18,19^. Finite element analysis also enabled scientific discoveries in knee biomechanics, particularly with recent developments in multiscale analysis, which provided the opportunity to infer chondrocyte deformations from knee joint simulations^20,21^. A contemporary summary of the utility of finite element analysis in knee biomechanics can be found in Kazemi et al.^22^.

Development of high fidelity models of the knee joint is a challenging task. A typical finite element analysis study requires imaging data (to reconstruct geometry), tissue mechanics data (to define material properties), and joint mechanics data (to evaluate model output); and if not available, appropriate adaptation of related model parameters from literature. When the information to build the model is available, many labor intensive and technically challenging procedures need to be completed to construct the model^8^: segmentation (to generate geometries), meshing (to discretize tissue volumes), constitutive modeling (to define mechanical response of tissues), model assembly, and verification and validation studies. After these steps, the model can be used for simulations aimed for understanding knee function in health and disease or for a-priori evaluation interventions, sometimes in a personalized manner. Studies describing development of knee joint models span almost two decades starting from early works such as that of Bendjaballah et al.^23^ to recent comprehensive models of Dhaher et al.^24^ and Kiapour et al.^13^. A PubMed search (http://www.pubmed.org, conducted on March 31, 2015) with the term “finite element AND knee” revealed 703 hits indicating a sincere level of in the community for modeling and simulation of the knee joint using finite element analysis. However, downloadable and reusable finite element representations of the knee are scarce. If and when available, such models can expedite generation of new knowledge of knee biomechanics and may facilitate clinical translation of new surgical strategies, therapeutic devices, rehabilitation protocols, and implants. Scientific investigators and clinicians can focus on using the available models rather than going through the long and painful steps of regenerating models.

## Goals

The goals of this study are threefold: (i) To describe Open Knee as an example of an open source and freely accessible finite element representation of the tibiofemoral joint, (ii) to provide use cases of Open Knee in order to illustrate various model customization and simulation workflows, which can assist scientific discovery and clinically relevant investigations in knee biomechanics, and (iii) to summarize the impact of Open Knee in the biomechanics community. In addition, this document is intended to provide the justification for community-driven and transparent development and dissemination of general purpose knee joint models where future efforts in modeling and simulation can focus on the generation of diverse, reliable, reusable, and accessible virtual representations of the knee joint to address pressing clinical and research problems.

## Model

**Figure 1:**
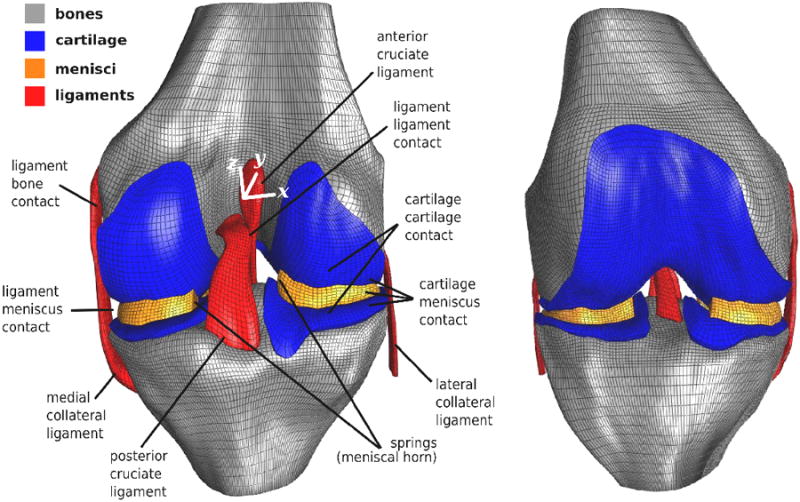
Open Knee provides a computational representation of tibiofemoral joint anatomy and mechanics. All model components are defined on the posterior view of the model, which can be seen on the left. The geometries of bones, cartilage, menisci, and ligaments were individualized to the specimen and discretized into meshes. An anterior view of the whole mesh can be seen on the right. Nonlinear mechanical properties of the tissue structures were based on information available in literature. For a given joint load, finite element analysis seeks for mechanical equilibrium which also resolves mechanical interactions between tissues, e.g., contact. In return, simulations predict of joint movements and tissue stresses and deformations. (Adapted from Open Knee(s) project site, https://simtk.org/home/openknee, courtesy of Ahmet Erdemir.)

Open Knee is a finite element representation of the tibiofemoral joint incorporating anatomy and mechanical properties of the joint's individual tissue structures and the mechanical interactions in between (Figure 1). The model and the development of it are described in detail in the Open Knee User's Guide^25^. Important aspects of the model and its development steps are summarized in here. The Open Knee User's Guide is also provided as a supplementary material to this document.

The tibiofemoral joint model was based on anatomical and mechanical data collected on a cadaver right knee from a 70 years old female donor (77.1 kg, 1.68 m)^26^. Anatomical images were acquired on a 1 Tesla magnetic resonance imaging machine (Orthone, ONI, Inc., Wilmington, MA) in axial, sagittal, and coronal planes, utilizing a 3D spoiled gradient echo sequence with fat suppression. Measurements of joint kinematics-kinetics under various laxity conditions (anterior-posterior translation, internal-external rotation, varus-valgus) and for combined loading scenarios were also conducted with the specimen loaded on a robotics testing system^26,27^ (Rotopod 2000, Parallel Robotics Systems Corp., Hampton, NH). The mechanical data set also includes gross measurements of anterior cruciate ligament length change, which was acquired with a DVRT (MicroStrain, Inc., Williston, VT) that was placed on the anteromedial bundle of the ligament. Anatomical and mechanical data collection procedures are described in more detail in Borotikar^26^.

Open Knee includes geometric representations of the bones (femur and tibia), cartilage (femoral and tibial), menisci (medial and lateral), and ligaments (anterior and posterior cruciate, medial and lateral collateral). These geometries were manually segmented from sagittal plane anatomical image set and represented by parametric surfaces. In following, hexahedral meshes of individual tissue structures were generated using TrueGrid (XYZ Scientific, Livermore, CA) to provide discretized representations of tissue volumes. The bones were modeled as rigid bodies and the rest of the tissues were modeled as deformable with nonlinear mechanical properties. Specifically, the cartilage is a nearly incompressible Neo-Hookean material^9^; the menisci is a Fung orthotropic hyperelastic material^28^ with horn attachments represented by springs^29^; and the ligaments are transversely isotropic hyperelastic materials (anterior and posterior cruciate ligaments^10^, medial and lateral collateral ligaments^30^). Contacts between tibial and femoral cartilage surfaces, between cartilage and menisci, between anterior and posterior cruciate ligaments were defined to represent mechanical interactions between the tissue structures during simulations of joint loading. To simulate joint loading, tibia is fixed in space and femur movements and loads can be prescribed by providing time histories of flexion angle and of forces and moments for the remaining degrees of freedom. A dynamic simulation (utilizing an implicit time integration) predicts joint movements and stresses and deformations of individual tissue structures under desired joint loads. All joint movements are described in an anatomically based coordinate system^31^. Simulations are conducted using FEBio^32^, a finite element analysis package specifically designed for biomechanics. FEBio is open source and freely available for academic research (http://febio.org/). Open Knee is compatible with FEBio version 1.3.0 and above. Interactive pre-processing (for model development) is possible by using PreView (> version 1.3.0) and interactive post-processing (for visualization of simulation results) can be conducted by utilizing PostView (> version 1.3.1). PreView and PostView are companion software to FEBio. Computer scripts were also developed to streamline pre- and post-processing steps in a programming environment. These allow customization of the model to reflect different loading and boundary conditions, tissue material properties, and permit calculation of desirable output metrics from simulation results, e.g., joint movements. Python programming language (http://www.python.org) was used for this purpose.

The model, the data (e.g., anatomical images) and the intermediate products (e.g., geometries, meshes, scripts) have been disseminated to public to maximize their utility for prospective modeling and simulation studies in biomechanics (see section on Dissemination). Licensing was based on Creative Commons Attribution-Share Alike (http://creativecommons.org/) to allow anyone to share and adapt the model for any purpose, even commercially, under the terms of attribution, e.g., by citing Open Knee, and share alike, e.g., by providing model extensions back to the public in a similar manner.

## Use Cases

In this section, a handful of use cases for Open Knee are presented. These studies utilized Open Knee to conduct various finite element analyses for the investigation of joint and tissue mechanics under different loading scenarios. The simulation cases illustrated the predictive capacity of Open Knee against passive joint motion data obtained from a sample population of cadaver knees^33^ and against tissue deformation data obtained for the specimen^26^, on which the Open Knee was based. Customization of the model to change joint loading conditions and to remove tissue components are also described. Such adaptations of the model has utmost importance to conduct clinically relevant simulations where predictions can assist evaluation of surgical interventions and therefore facilitate clinical decision making. Training opportunities for physicians, scientists, and engineers can also be realized where a comprehensive understanding of the function of the knee and its tissue substructures in an interactive fashion may be possible.

### Joint Kinematics during Passive Flexion

The primary goal of this use case was to conduct a baseline simulation with Open Knee where the capacity of finite element analysis for simultaneous predictions of joint movements and tissue deformations was illustrated. Such predictions should be evaluated at multiple levels, not only for Open Knee but for any knee model. For example, the tibiofemoral joint movements under various loading conditions should be inline with knee population data (Does the model behave similar to the knees described in literature?) and with specimen-specific response (Does the model behave similar to the knee on which it was based on?). Similarly, predictive power of the model to estimate tissue level mechanical metrics, e.g., contact pressures and forces, ligament forces, meniscus deformations, need to be assessed. This use case additionally focused on the evaluation of the performance of Open Knee for prediction of coupled tibiofemoral joint movements, which were assessed against passive flexion data previously collected on cadaver knee specimens^33^.

**Figure 2:**
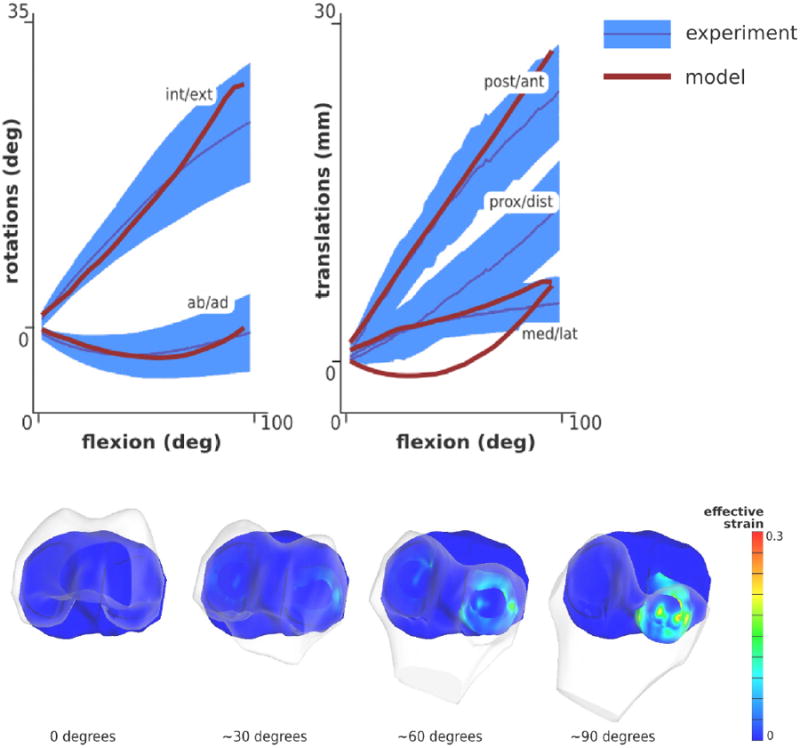
Open Knee was used to simulate passive movements of the joint by prescribing tibiofemoral flexion and setting the remaining degrees of freedom free. Simulation predictions reasonably agreed with movement data acquired on a sample of cadaver knee specimens^33^. Internal/external rotation and abduction/adduction, as represented in a joint coordinate system^31^, illustrated the expected coupling of knee joint movements (top panel). Predicted translations, i.e., the displacement of the posterior tibial insertion of anterior cruciate ligament relative to femur, matched those measured for the cadaver knees for flexion angles of up to 50°, except for proximal/distal movement. Visualization of joint movement confirmed the model's deviation from measured joint behavior, particularly for internal/external rotation at high flexion angles. This possibly caused unrealistic deformations of the lateral cartilage and menisci (bottom panel). This use case illustrates the premise of Open Knee for prediction of joint movements and tissue stresses and strains, all as a function of a desired joint loading profile. (Adapted from Open Knee(s) project site, https://simtk.org/home/openknee, courtesy of Ahmet Erdemir.)

Wilson et al.^33^ tested 15 cadaver knee specimens by fixing the tibia and flexing the femur up to 100° during which the remaining translations and rotations of the joint were set free, therefore, guided by the passive flexion of the femur. The tibiofemoral joint model was also subjected to the same loading conditions. The predicted rotations of the femur were described using an anatomically based joint coordinate system^31^, which was also used to report measurements on the cadaver specimens^33^. The displacements of the posterior tibial insertion of the anterior cruciate ligament relative to the femur provided translations obtained from simulations to directly compare against experimental data^33^. The overall agreement between model predictions and experimental measurements of movements were reasonable (Figure 2) given many uncertainties that were not accounted for, e.g., alignment of model and experimental coordinate systems aka registration, lack of in situ ligament strain^24^, literature based material properties, etc. Discrepancies were particularly emphasized for proximal-distal translation throughout the whole passive flexion. The model overpredicted internal rotation of the tibia and posterior translation of the tibial insertion of the anterior cruciate ligament, particularly for high flexion angles. This issue possibly caused elevated deformations of the lateral cartilage and the lateral meniscus at high flexion angles (Figure 2).

Simulations as part of this use case rendered Open Knee as a virtual tool exhibiting coupled movements of the tibiofemoral joint during passive flexion – a fundamental property of knee mechanics^33^. It should be noted that evaluation of complete joint movements (all translations and rotations) in finite element analysis of the tibiofemoral joint is rare, with a few exceptions^11,13^. Adequate prediction of joint response does not necessarily indicate appropriate estimations of tissue stresses and strains. Nonetheless, the combined effect of individual tissue response on joint mechanics will be appropriate to evaluate the overall knee response virtually. From a training perspective, this model and the simulation case can provide opportunities where students of engineering and scientific disciplines and clinical fellows can explore individual role of major joint structures on coupled movements of the knee. From a clinical perspective, simulations can indicate the influence of surgical interventions on passive joint movements, where the surgeries can be represented by adapting the model, e.g., to accommodate removal of tissue structures, changing of ligament mechanical properties and their insertion areas.

### Passive Flexion after Meniscectomy

**Figure 3:**
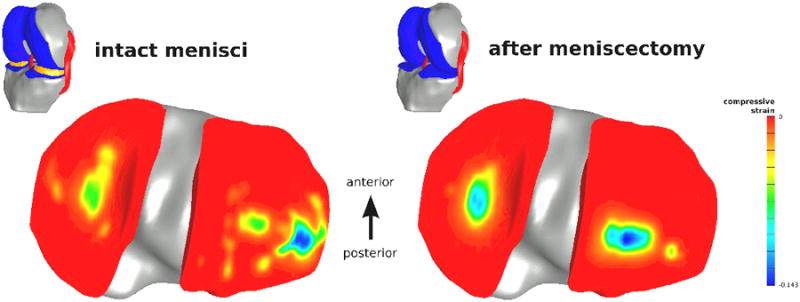
Simulation of meniscectomy indicates customization potential of the Open Knee to explore clinically relevant interventions. The tibifemoral joint was flexed to 45° in a passive manner; a compressive load of 100 N was applied and the remaining rotations and translations of the joint were set free. Comparison of the predictions when using the model with intact menisci and after removal of the menisci revealed increased compression of the tibial cartilage both for the medial and lateral sides where it engaged with the femoral cartilage. After meniscectomy, contact location on the lateral side moved posteriorly. This was attributed to the increased internal rotation of the tibia after resection of the menisci. (Adapted from Open Knee(s) project site, https://simtk.org/home/openknee, courtesy of Ahmet Erdemir.)

This use case focused on the utility of Open Knee to simulate the mechanical effects of a clinical intervention on the tibiofemoral joint movements and cartilage stresses and deformations. A virtual meniscectomy was carried out by removing the representations of both medial and lateral menisci in the model. First a simulation with the menisci intact model was conducted to predict joint movements and tissue deformations for prescribed flexion angles of up to 45°. A 100 N compressive force was applied on the femur and the translations and rotations of the bone were set free. Then, a simulation with the model after meniscectomy was carried out. As anticipated, when the menisci were intact, the contact area included areas under the footprint of menisci, particularly for the lateral meniscus (Figure 3). After removal of the menisci, the contact regions were concentrated at the central region of tibial and medial plateau where femoral and tibial cartilage oppose each other (Figure 3). At these regions, the deformations of the cartilage increased, particularly for the lateral side, indicating a larger load transmission shifted towards these contact areas. The contact location on the medial site remained at a similar position as in the intact knee whereas the lateral contact region shifted posteriorly. Further evaluation of joint movements before and after meniscectomy revealed that the internal rotation of the tibia increased from 6° to 11° as a result of the surgical operation.

In this simulation case study, the premise of Open Knee for evaluation of the impact of clinical interventions was established. Finite element analysis of meniscectomy not only indicated potentially hazardous effect of the surgery on other tissue structures, e.g., increased mechanical loading of cartilage, but also portrayed changes in the overall mechanical function of the joint. In a sense, Open Knee can be utilized as a virtual prototyping tool to explore new strategies to improve surgical outcome, both in terms of maximizing functional performance and minimizing undesirable secondary effects.

### Anterior Cruciate Ligament Deformations

**Figure 4:**
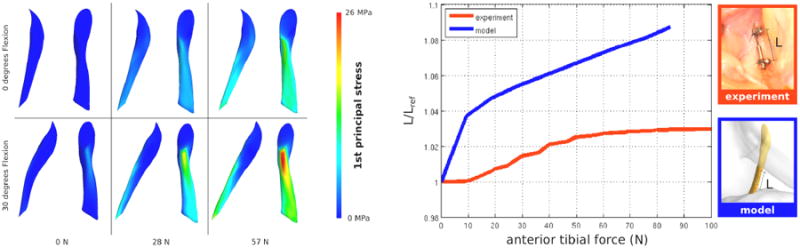
Open Knee can be used to understand mechanical function of tissue structures under varying joint loading conditions. In this use case, anterior tibial forces were applied at flexion angles of 0° and 30° to explore mechanics of the anterior cruciate ligament. First principal stresses within the ligament are displayed for different force loading levels (left panel, including sagittal and anterior views of the ligament). Anterior bundle of the ligament was primarily loaded; larger forces and higher flexion angle resulted in increased tissue stress. Gross deformations of the anterior cruciate ligament were also measured on the specimen used for the development of Open Knee^26^. While there was a lack of absolute correspondence between ligament elongations observed during experiments and predicted by simulations (right panel, 0° flexion results are shown), the similarities in relative relationships between ligament deformations and anterior tibial loads were encouraging for prospective uses of the model. (Adapted from Open Knee(s) project site, https://simtk.org/home/openknee, courtesy of Ahmet Erdemir.)

In this use case, the utility of Open Knee to understand tissue mechanical function in relation to joint loading was explored. The anterior cruciate ligament was the tissue of interest and its response, when the joint was loaded with anterior tibial forces, was predicted by various simulations. The model was set at a prescribed flexion of 0°; the tibia was fixed and posteriorly directed forces up to approximately 100 N were applied on femur to simulate an anterior drawer test on the joint. In another simulation, this loading scenario was applied after moving the joint to 30° flexion. The 1^st^ principal stresses in the anterior cruciate ligament were quantified to evaluate longitudinal loading exhibited by this tissue structure (Figure 4). The anterior portion of the ligament exhibited larger stresses, which increased as a function of joint force. At 30° flexion, the stresses were higher (Figure 4). For the same knee on which Open Knee was based, mechanical testing data representative of anterior drawer were also available^26^. This data set included joint movements (described in an anatomically based joint coordinate system^31^) and gross measurements of anterior cruciate ligament length (acquired with a DVRT; MicroStrain, Inc., Williston, VT), all collected during anterior-posterior laxity testing. Measurements recorded by the DVRT were compared against model predicted length change to provide specimen-specific evaluation of simulation predictions at the tissue level. The DVRT was inserted at the distal end of the ligament approximately 3 mm above the tibial insertion and at the proximal end approximately 11 mm proximal to distal insertion point^26^. Model predicted anterior cruciate ligament length utilized the distance between two mesh nodes approximating this placement (Figure 4). The anterior cruciate ligament length increased monotonically as a function of anterior tibial force, both in the experiments and for the model (Figure 4). In an absolute sense, model predictions were higher. Interestingly, for 0° flexion, predicted anterior translation of the joint was lower than the measured anterior translation (∼5 mm vs ∼ 7 mm at 80 N). At this flexion angle and at that anterior load, the cadaver specimen exhibited almost no internal rotation of the tibia, whereas in the model, tibia rotated internally by almost 8°. Albeit lower anterior translation of the joint, the higher internal rotation of the tibia likely positioned origin and insertion of the ligament such that model predicted anterior cruciate ligament lengths were higher. It should be noted that comparisons between model and experiment suffered from uncertainties in aligning coordinate systems between in vitro and in silico representations. Nonetheless, the simulations illustrated the coupled mechanical function of the anterior cruciate ligament on anterior forces and the resulting translation and rotation of the joint.

Simulations of anterior drawer tests portrayed the possibility to use Open Knee to evaluate tissue function by adaptation of the model to apply desired loading situations. This simple study also emphasized the need for holistic evaluation of tissue mechanics incorporating interpretation of complete joint movement predictions rather than focusing on the dominant loading direction. When conducted with this mindset, finite element analysis will be instrumental for scientific understanding of knee biomechanics and clinical care of the tibiofemoral joint.

## Enabled Scientific Studies and Clinically Oriented Simulations

Development and evaluation of computational models, particularly of those aimed for finite element analyses of musculoskeletal joints, are labor intensive processes requiring comprehension and application of advanced engineering principles. When available, such models can disrupt existing barriers for routine use of simulations for scientific discovery and for clinical translation. Specific to knee joint biomechanics, open source and freely available models can empower the community with opportunities to learn knee function. In addition, with the redirected focus on model customization (rather than its reproduction from scratch), others can immediately conduct simulations for hypothesis generation and for intervention testing. Based on this premise, Open Knee was released in 2010 and since then it has been downloaded by the community for more than 500 times. Feedback provided by the users indicated diverse intentions for downloading the model ranging from research and training purposes to the desire to conduct clinically relevant simulations. A handful of investigators already completed such studies, where Open Knee facilitated the modeling and simulation workflow and therefore expedited delivery of new scientific knowledge and clinical guidance.

Open Knee and the intermediate components of the model were used in scientific studies that aimed for development of finite element analysis techniques and for in-depth examination of tissue function. Articulating tissue geometries, provided by Open Knee, served to test biphasic contact formulations in a physiological joint representation^34^. These geometries also expedited development of another model, which was used to simulate and interpret time-dependent contact mechanics of the tibiofemoral joint during application of loads at a level of body weight^35^. Open Knee model, in its disseminated form, was simulated under a compressive load of one body weight and simulation results were used in a multiscale analysis workflow to predict chondrocyte mechanics in the tibial and femoral cartilage as a function of joint load^20^. Such joint level simulations, using Open Knee in conjunction with FEBio^32^, were conducted by others as well, to understand mechanics of the cartilage^36^. Derivative models of Open Knee has also been developed^37^, by porting the model to Abaqus (SIMULIA, Johnston, RI) and adding the patellofemoral joint.

Open Knee also enabled studies with direct clinical relevance. Westermann et al. utilized the mesh as a springboard for finite element analysis of anterior cruciate ligament reconstruction^38^. Their study indicated that larger graft size not only influenced the stresses in other structures (lower menisci stresses and cartilage contact pressures) but also decreased joint laxity during a Lachman test. Majority of clinically oriented studies that benefited from the open source tibiofemoral joint representation focused on meniscus problems and their management. Mechanics of the medial meniscus during collisions were explored by simulating Open Knee with different loading and boundary conditions^39^. Meng et al. used cartilage and meniscus geometries to understand changes in time-dependent contact between articular surfaces as a result of meniscectomy^35^. Similar to the use case reported above, they observed increased compressive stress in the cartilage in the meniscectomized knee. Explorations of meniscectomy using Open Knee geometries have been extended by Luczkiewicz et al.^40^, who studied the mechanics of radial posterior meniscus root tears (partial or complete) and unilateral meniscectomy. Additional simulations aimed at virtual prototyping of meniscal implants^41^. This latter study identified the importance of implant size (not shape) on cartilage contact with smaller implants reducing contact pressures^41^.

## Discussion

This study described Open Knee, an open source and freely available model of the tibiofemoral joint. To the authors knowledge, this model was the first and only finite element representation of the knee made available to the public at the time of its initial dissemination. Despite its limited performance, the model and its intermediate components were reused by many investigators (other than the developers) to answer scientific questions and to depict the role of surgical interventions and implants on the mechanics of the tibiofemoral joint and its tissue structures. It is believed that the availability of Open Knee and relevant data associated with it expedited studies conducted by third-party investigators by eliminating the need to develop a virtual representation of the knee from scratch. The model therefore complements the simulation software and computing hardware infrastructure necessary for in silico explorations in knee biomechanics.

The use cases of Open Knee provided many prospective opportunities for the model's utilization for scientific quests and for clinically relevant computational studies. These simulations provided a range of scenarios that can be implemented as different loading and boundary conditions, e.g., passive flexion vs anterior drawer, and adaptations of the model, e.g., removal of menisci. The variety of output metrics indicated the possibility for assessment of knee mechanics at the joint level, i.e., through examination of predicted joint movements, and at the tissue level, e.g., cartilage and ligament stresses and strains, in a wholesome manner.

The use cases also established the bounds of Open Knee's predictive capacity. While coupling of passive joint motions is apparent in Open Knee's response, the model seems to exhibit rather compliant behavior for internal rotation of the tibia. This situation was observed for high flexion angles during passive joint motion and also when an anterior drawer load was applied. The material properties of Open Knee were not specimen-specific, rather, they were approximated from information available in literature. If one uses different material properties, model's emergent joint mechanics response may change, in a desirable manner or not. Incorporation of in situ ligament strain^24,30^ may help establish improved realism of mechanical joint response. Similarly, addition of previously neglected tissue structures or interactions, may help stabilize the joint in accordance with the expected specimen-specific and population response. For example, modeling a transverse ligament connecting anterior regions of the menisci may help stabilize the lateral meniscus. Defining connectivity between medial collateral ligament and the medial meniscus may serve a similar purpose this time on the opposite side.

At its current state, Open Knee remains to be a useful computational tool for any interested party. Novice or expert, anyone can involve in computational biomechanics of the knee without investing large amounts of time and effort for model development. Enhancements are certainly needed in order to increase the model's fidelity and its credibility. Yet, with the Open Knee philosophy, implementation of these improvements are not exclusive to the developers but to anyone who may be interested in.

## Future Generation of Models

Open Knee experience, current state-of-the-art in simulation-based approaches in knee biomechanics, and the immediate need for routine and reliable utilization of modeling to address challenging problems in clinical care of the knee indicate many gaps to be filled with the development and availability of next generation knee models. Virtual knees authentic to specimen-specific anatomical and mechanical information, all acquired in vitro, are scarce. In many simulation studies, specimen-specificity and patient-specificity commonly imply individualization to the anatomy of the knee^42^. Mechanical properties of the tissues were commonly adapted from literature or at best tuned to match the model's joint level response to the specimen's laxity response^43^. To accomplish complete specimen-specificity, anatomical data (from which tissue geometries are obtained) and tissue characterization (from which tissue material properties can be extracted) should be collected on the same specimen, on which the model will be based on. Use of such data sets will minimize uncertainties not only associated with anatomical representation but also with assignment of mechanical properties^24^. In following, required fidelity for patient-specific modeling can be evaluated appropriately. Specimen-specificity is also needed for assessment of predictive capacity. Recent modeling efforts conducted comprehensive validation of the knee model by comparing its output for joint movements to data collected on a sample population of cadaver knees^13^. While these elaborate activities increase the credibility of the model, without specimen-specific comparisons, it is not possible to assess whether the mismatches are a result of modeling errors or due to natural variations in population response. Similarly, various error sources may negate each other resulting in a match between simulations and variability within experimental data.

The need for building virtual populations for probabilistic studies and for in silico clinical trials is emerging. In response, the computational biomechanics community has started developing virtual knees representative of anatomical variations^42^. Nonetheless, the range of potential clinical populations may be wide, for example, requiring models of young knees that may be exposed to trauma and models of elderly knees that may be subject to natural progression of cartilage degeneration and osteoarthritis. These models should accommodate not only population-dependent anatomical properties but also alterations in tissue material properties. With a large group of virtual knees founded on comprehensive data, it may be possible to provide simulation-based training opportunities to prepare physicians to natural variations in knee mechanics and to conduct in silico trials to effectively evaluate the robustness of an intervention.

Knee biomechanics community can significantly benefit from an open development modeling approach where next generation models can be built with community input. This is the next step to open source and free model sharing, where input from clinicians (potential users) and engineers (potential developers) can be acquired not after but during the model development and testing procedures. In following, opportunities for crowd-sourcing, collaboration possibilities, enhanced repeatability and reproducibility of simulations will be available for reliable and effective translation of models to practice. Combined with user-centered development, i.e., increasing usability of the models, and by exploiting cloud computing, simulations using virtual knees can be accessible to the community at large, irrespective of the user's background.

The goal for future is to advance computational knee biomechanics through a platform for community driven modeling and simulation. With the anticipated progress from Open Knee to Open Knee(s) – Generation 2, models of the knee joint will become indispensable and routine tools of scientific conduct in knee biomechanics and for clinical management of knee disorders.

## Dissemination

Open Knee (Open Knee(s) – Generation 1), including its intermediate components (data, geometry, mesh, scripts, model), is freely available in the “Downloads” section of the project site – https://simtk.org/home/openknee. Prospective data and models, generated as part of the Open Knee(s) – Generation 2 initiative can also be accessed at that section. Customized models and simulation results described in this study are available through relevant folders of the “Source Code Repository” at the project site. Those interested in the development of next generation knee models are encouraged to browse the “Source Code Repository” and particularly the “Wiki” at http://wiki.simtk.org/openknee, where roadmap, specifications and work in progress can be accessed.

## Supplementary Material

User’s Guide
