# Supplementary material for: Open Knee: Open Source Modeling & Simulation to Enable Scientific Discovery and Clinical Care in Knee Biomechanics
Source: J Knee Surg. Author manuscript; Available in PMC 2016 May 23. (PMC4876308; doi:10.1055/s-0035-1564600)
Supplement: User’s Guide [file NIHMS778594-supplement-User_s_Guide.pdf]

Open Knee: A Three-Dimensional Finite Element Representation of the Knee Joint

- User's Guide -

- Version 1.0.0 -

prepared by

Ahmet Erdemir & Scott Sibole

Computational Biomodeling Core & Department of Biomedical Engineering

Lerner Research Institute, Cleveland Clinic

December 17, 2010

cc BY SA

Table of Contents

Summary....3
Distribution Files and Folder Structure....3
Quick Start....4
- Running Open Knee....4
- Changing Open Knee....4

Software Compatibility....6
Data....6
- Specimen....6
- Magnetic Resonance Imaging....6
- Geometry....7
- Mesh....9

Tools....12
- Mesh Generation....12
- Model Generation....12

Model....13
- Geometry & Mesh....13
- Material Properties....15
- Interactions....18
- Other Components....19
- Loading & Boundary Conditions....20
- Output....20

Case Simulations....21
- Solver Software....21
- Simulation Settings....22
- Case 1: Passive Knee Flexion....22

Verification and Validation....23
- Solver Verification....23
- Sensitivity to Simulation Settings....23
- Mesh Convergence....24
- Repeatability & Reproducibility....24
- Validation....25
- Sensitivity to Model Parameters....25

Known Limitations....25
Citing Open Knee....27
Access and Contact Information*....27
- Website....27
- Wiki....27
- Repository....27
- Forums....27
- Correspondence....27

Acknowledgments....28
References....29
Licensing....31
Trademarks and Ownerships....31

Summary

The goal of the Open Knee project is to provide a freely available and open source finite element representation of the knee for predictive simulations of joint loading and its effects on the underlying tissue structures. The model, as presented in here, is a work in progress and distributed as is. It can be utilized for learning finite element analysis in joint biomechanics; to understand the mechanical function of the knee and its components; and pending verification and validation, for scientific research on the mechanics of the joint as a whole and of its individual tissue structures. The users are expected to consult on the 'Known Limitations' section to evaluate the capacity of the model for their use. This document describes how to use the model, i.e. conduct simulations with different loading conditions, and also details the underlying development efforts for those interested in customizing the model for their needs or to help increase confidence in model predictions by addressing known limitations, and performing sensitivity and validation simulations. For bleeding edge information on Open Knee, please refer to https://simtk.org/home/openknee.

Distribution Files and Folder Structure

```
openknee-v1.0.0.zip
./
├── dat
│   ├── geo
│   ├── mri
│   └── msh
├── doc
│   └── guide.pdf
├── license.txt
├── readme.txt
├── sol
│   └── passive_flexion.zip
└── src
    ├── abq2feb.cnfg
    ├── abq2feb.py
    ├── tf_joint.feb
    └── tf_joint.tg
```

Quick Start

All data files, model development scripts and the model file are accessible after uncompressing the distribution file. Due to referencing of relative paths in several scripts, it is important to maintain the file and folder structure present in the compressed distribution file.

Running Open Knee

The model, with loading and boundary conditions simulating passive knee flexion, is available in the following location:

<PATH_TO_UNCOMPRESSED_DISTRIBUTION>/src/tf_joint.feb

To be able to execute a simulation with this model, one must first download and install FEBio, a freely available finite element analysis software, from http://mrl.sci.utah.edu/software/febio. Once FEBio is installed, in command prompt (or terminal window) one can navigate to the location of the model file, and start a simulation by executing the command:

febio -i tf_joint.feb

febio is the executable to run finite element analysis; it may be different depending on the operating system, e.g. *febio.lnx*. The user needs to make sure to add the location of this executable to the path of their operating system so that it can be executed in any directory. Successful completing of the simulation will result in two files in the same folder: tf_joint.plt and tf_joint.log. Results can be visualized in PostView, post-processor for FEBio, by opening the tf_joint.plt file. PostView can be downloaded from http://mrl.sci.utah.edu/software/postview.

Changing Open Knee

There are multiple ways to make changes to the Open Knee model. The model file can be updated by using PreView, pre-processor for FEBio. The model file is in XML format therefore allowing users, who have knowledge of FEBio syntax and enjoy the pain, edit it directly using a text editor. For advanced users comfortable with running scripts, a Python script is also included in the distribution to automate model generation for FEBio. An alternative approach is to import the text-based mesh file into PreView or into pre-processing software for other finite element analysis packages and define remaining model components from scratch. Neither of these methods allow changes in geometry and mesh at this moment. Such alterations need to be conducted a-priori.

Editing Model with PreView

Requirements: PreView, available from http://mrl.sci.utah.edu/software/preview

Files:

<PATH_TO_UNCOMPRESSED_DISTRIBUTION>/src/tf_joint.feb

Once PreView is installed, the user can import the model file (tf_joint.feb) and change the desired components of the model, e.g. material properties, contact, boundary conditions, etc, through a graphical interface. The new model should be saved in FEBio format to run as described above.

Editing Model with Python Script

Requirements: Python (minimum version 2.6.5) & SciPy (http://www.scipy.org)

Files:

<PATH_TO_UNCOMPRESSED_DISTRIBUTION>/src/abq2feb.py

<PATH_TO_UNCOMPRESSED_DISTRIBUTION>/src/abq2feb.cnfg

The users are encouraged to employ the Python script since it bypasses tedious pre-processing steps and the potential for errors associated with it. This approach assumes that Python and SciPy is installed on the user's computer. On Linux systems this can be done by using software repository install features or if preferred, through a manual install. On other operating systems, the user may download each software individually or get compiled packages, which include Python, SciPy, and various interpreters wrapped in an installer, e.g. Python(x,y) (http://www.pythonxy.com). Changing the model using this approach involves updating the configuration file (abq2feb.cnfg), executing the Python script (abq2feb.py) with this configuration, which in turn generates an FEBio file for the updated model:

- *Configuration* (abq2feb.cnfg) – Configuration file includes specifications of many model attributes: output model name, path of the mesh file (text based Abaqus input file format, *.inp), solver/time settings, material properties, interactions, loads, boundary conditions, and anatomical locations for transformation to a physiological coordinate system. Execution of the python script with the supplied configuration file will generate the distributed model file, tf_joint.feb, by overwriting it. Keywords in the configuration file are identified by '#' and comments by '##'.
- *Execution* (abq2feb.py) – To execute the script, the user needs to navigate to the directory containing the script and the configuration file (by default abq2feb.cnfg) in the command prompt. In following, execution of the following command will generate a model file using the output model name defined in the configuration file:

  python abq2feb.py -- abq2feb.cnfg

  One can substitute the configuration file. In Linux systems, it may be necessary to omit the '--'.
- *Output* (*.feb) – Execution of the Python script results in a FEBio model file reflecting the content of the configuration file. The filename will be based on the model entry in the configuration file. By default, the distribution results in tf_joint.feb in the same folder as the script and the configuration file.

Editing Model from Mesh

Requirements: Pre-processing software capable of importing text-based Abaqus input files

Files:

<PATH_TO_UNCOMPRESSED_DISTRIBUTION>/dat/msh/tf_joint.inp

To use any desired finite element analysis package, the model can be generated from scratch, starting with the mesh file (tf_joint.inp). The mesh file is based on Abaqus input file format, which is text based and can be imported to a variety of finite element analysis software. The

mesh file also includes a wide range of node, element, and surface set definitions to facilitate definition of material boundaries, contact interfaces, etc. One can import this file into PreView to go through all model definition steps in a graphical interface or to another package such as Abaqus if it's the users preferred finite element analysis platform.

Software Compatibility

Current Open Knee model was tested and found compatible with PreView version 1.3.0, FEBio version 1.3.0.1428, and PostView version 1.3.1. PreView is a pre-processing software for finite element analysis solver FEBio. PostView is the post-processor for the same solver. All these packages can be accessed through http://mrl.sci.utah.edu/software. The mesh script is compatible with TrueGrid 2.2.6 but NOT later versions i.e. 2.3.4.

Data

Specimen

Table 1. Specimen details.

**NDRI ID**	89956*
**MRMTC#**	022508-03#
**Side**	Right
**Donor Age**	70 years
**Donor Estimated Body Weight**	170 lbs (77.1 kg)
**Donor Height**	5'6" (1.68 m)
**Donor Gender**	Female
**Donor Cause of Death**	Pneumonia/Cancer

*This is the specimen identification number from National Disease Research Exchange, where the specimen was acquired.

#This is the specimen identification number from Musculoskeletal Robotics and Mechanical Testing Facility at the Cleveland Clinic, where mechanical testing of the specimen was performed.

Magnetic Resonance Imaging

All MRI data is located in the following folder in widely accessible DICOM format:

<PATH_TO_UNCOMPRESSED_DISTRIBUTION>/dat/mri

The knee specimen was imaged at the Biomechanics laboratory of the Cleveland Clinic using a 1.0 Tesla extremity MRI scanner (Orthone, ONI Medical Systems Inc, Wilmington MA). A scanning protocol that provided a reasonable contrast for articular cartilage and ligaments within the same scan were used [1] (Figure 1). The specifics of the protocols can be found in Table 2.

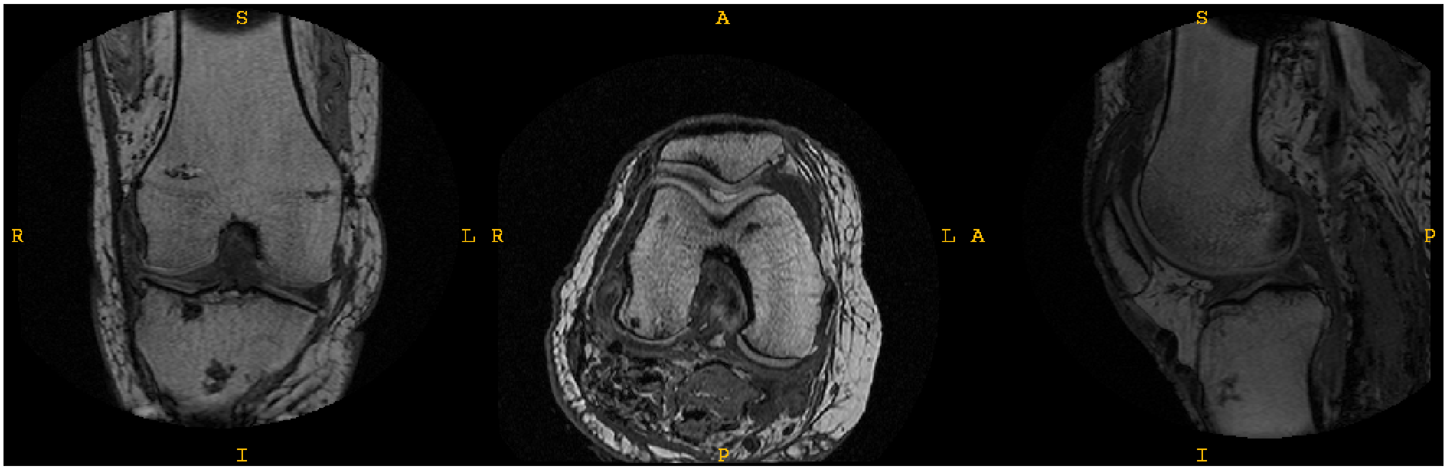

Figure 1. Magnetic resonance imaging of the knee.

During the scans, the knee was kept in full extension position. In summary, the imaging technique utilized a 3D spoiled gradient echo sequence with fat suppression, TR = 30, TE = 6.7, Flip Angle = 200, Field of View (FOV) = 150mm X 150mm, Slice Thickness = 1.5mm. Scans in three anatomical planes; axial, sagittal, and coronal, were conducted. Total scanning time was approximately 18 minutes. These specific sequence parameters produced images that highlighted articular cartilage such that it could be easily differentiated from surrounding bone and tissue. The protocols and the image set reflect partial data from the doctoral work of Bhushan Borotikar [1].

Geometry

Geometries of tissue structures representative of knee anatomy are available in IGES format:

<PATH_TO_UNCOMPRESSED_DISTRIBUTION>/dat/geo

In this distribution of the Open Knee, the knee geometry relies on manual segmentation obtained from sagittal MR images. VolSuite (http://www.osc.edu/archive/VolSuite/) was used for this purpose. This initial geometry set was generated by Craig Bennetts of the CoBi Core at the Cleveland Clinic. Three-dimensional spline curves were generated using SALOME (http://www.salome-platform.org), which were later used to develop NURBS surfaces using the loft feature in the geometric modeling package Rhinoceros (McNeel North America, Seattle, WA) (Figure 2). Due to poor visibility of the lateral collateral ligament in sagittal image sets, its geometry was approximated. Geometries are provided in a coordinate system aligned with the first sagittal MR image (all units in millimeters):

- origin - top-left corner
- x-axis - pointing towards right (anterior to posterior)
- y-axis - pointing downwards (superior to inferior)
- z-axis - pointing inwards (medial to lateral)

Table 2. Settings for magnetic resonance imaging.

**Scan Parameters**	Sagittal	Axial	Coronal
Pulse Sequence	GE3D	GE3D	GE3D
TR	30	30	30
TE	8.9	8.9	8.9
Frequency	260	260	260
Phase	192	192	192
FoV	150	150	150
BW	20	20	20
Echo Train	1	1	1
NEX	1	1	1
Flip Angle	35	35	35
Time	5.03	3.19	3.30
**Scan Options**	Sagittal	Axial	Coronal
Graphics SL	Y	Y	Y
RF spoiling	Y	Y	Y
Fat Suppression	N	N	N
Minimum TE	Y	Y	Y
Inversion recovery	N	N	N
Partial data	N	N	N
No phase wrap	Y	Y	Y
Spatial saturation	N	N	N
Flow comp	N	N	N
Magnetic transfer	N	N	N
**Prescan Parameters**	Sagittal	Axial	Coronal
Prescan	Auto	Auto	Auto
Center freq.	Peak	Peak	Peak
**Slice Parameters**	Sagittal	Axial	Coronal
Number of slices	70	45	60
Slice thickness (mm)	1.5	1.5	1.5
Gap (mm)	0	0	0
Range (mm)	105	67.5	90

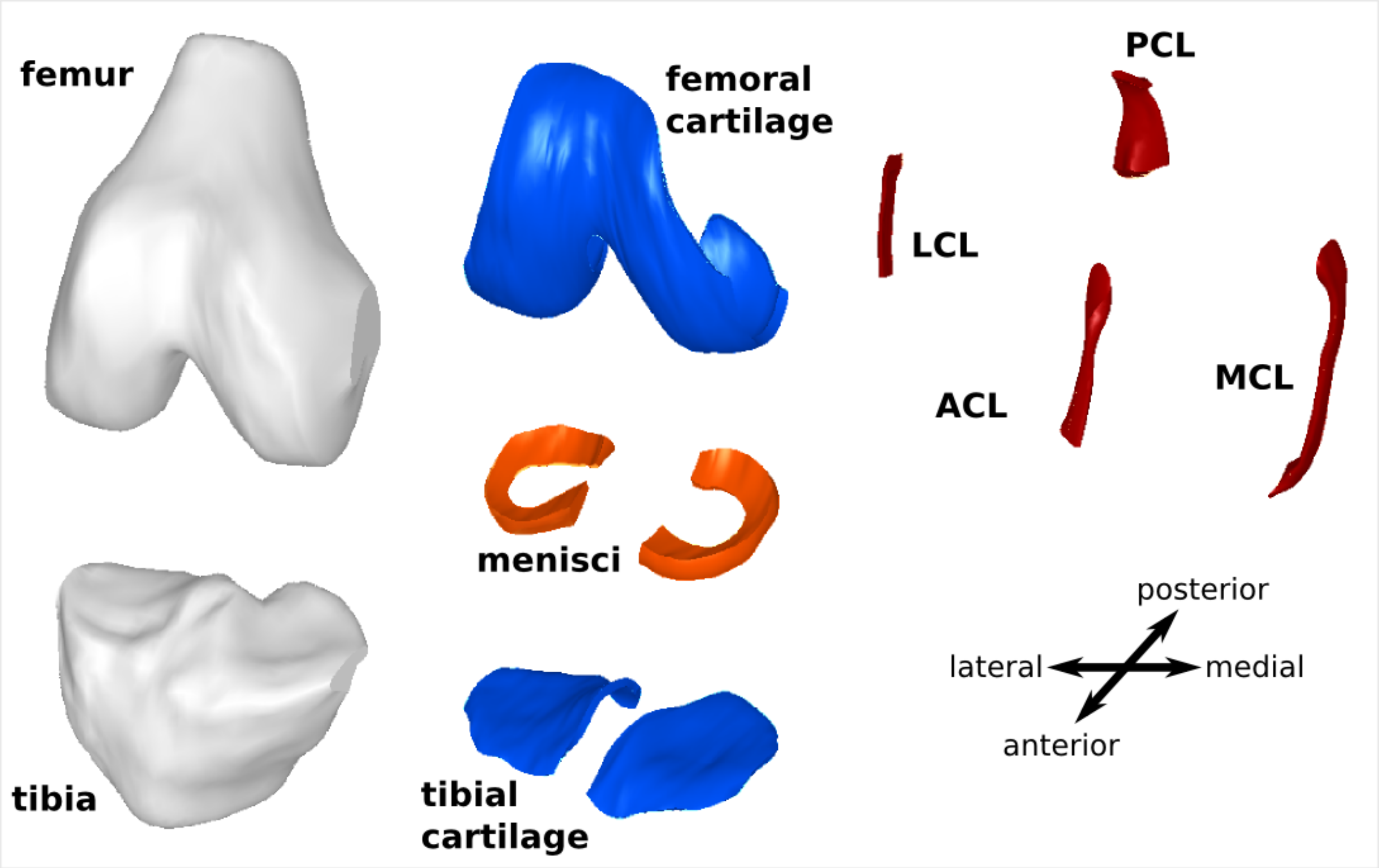

Figure 2. Solid models of the bones, cartilage, ligaments, and menisci.

Following geometric models are available to represent various knee structures:

- femur
- tibia
- femoral cartilage
- tibial cartilage
- medial collateral ligament
- lateral collateral ligament
- anterior cruciate ligament
- posterior cruciate ligament
- medial meniscus
- lateral meniscus

Mesh

Mesh of the tibiofemoral joint and its structures are available in the following location as a text file conforming mesh definition conventions of Abaqus (Simulia, Providence, RI):

<PATH_TO_UNCOMPRESSED_DISTRIBUTION>/dat/msh/tf_joint.inp

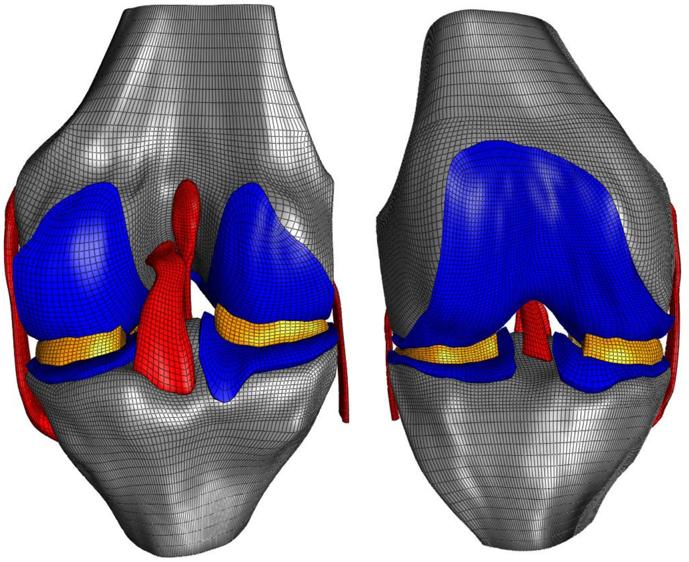

Figure 3. Mesh of the knee structures.

The mesh was generated using TrueGrid software (XYZ Scientific, Livermore, CA) based on the geometric models of tibiofemoral joint tissue structures (see Mesh Generation section in Tools). The file provides node definitions in the same coordinate system as the geometries in millimeters. 56433 hexahedral elements define connectivity between nodes for the soft tissue structures, and 25220 quadrilateral shell elements represent discretized bone geometry (Figure 3). This file can be imported into a variety of finite element analysis pre-processing packages to develop the knee model. A large group of node, element, and surface sets are also available in the file to facilitate definition of material boundaries and contact as well as loading and boundary conditions:

- Node Sets
    - Rigid interface nodes for femoral cartilage to femur attachment - *f2fem*
    - Rigid interface nodes for tibial cartilage to tibia attachment - *tc2tib*
    - Rigid interface nodes for ligament to femur attachment - *femlig*
    - Rigid interface nodes for ligament to tibia attachment - *tiblig*
    - anterior-lateral meniscal horn - *lmant*
    - posterior-lateral meniscal horn - *lmpost*
    - anterior-medial meniscal horn - *mmant*
    - posterior-medial meniscal horn - *mmpost*
- Element Sets
    - femur - *femur*
    - tibia - *tibia*
    - femoral cartilage - *fcart*
        - femoral cartilage without patella region - *fcartr*
        - deep zone - *fcartb*
        - transitional zone - *fcartm*

    - superficial zone - *fcartt*
  - tibial cartilage - *tcart*
    - deep zone - *tcartb*
    - transitional zone - *tcartm*
    - superficial zone - *tcartt*
  - medial meniscus - *med meni*
  - lateral meniscus - *lat meni*
  - medial collateral ligament - *mcl*
    - anterior - *amc*
    - middle - *mmc*
    - posterior - *pmc*
  - lateral collateral ligament - *lcl*
    - anterior - *alc*
    - middle - *mlc*
    - posterior - *plc*
  - anterior cruciate ligament - *acl*
    - anterior - *aac*
    - posterior - *pac*
    - femoral insertion region (fibers defined by different nodal numbering due to need for different meshing technique) - *aclfiber*
  - posterior cruciate ligament - *pcl*
    - anterior - *apc*
    - posterior - *ppc*
- Surface Sets
  - femoral cartilage - *fcs*
    - femoral cartilage without patella region - *fcsr*
  - tibial cartilage - *tcs*
  - femur surface contacting mcl - *femmcl*
  - femur surface contacting lcl - *femlcl*
  - tibia surface contacting mcl - *mcltib*
  - tibia surface contacting lcl - *lcltib*
  - mcl - *mcl_surf*
    - only lateral side - *mcls*
  - lcl - *lcl_surf*
    - only medial side - *lcls*
  - acl - *aclsurf*
  - pcl - *pclsurf*
  - lateral meniscus surface contacting tibial cartilage - *lmtib*

- lateral meniscus surface contacting femoral cartilage - *lmfem*
- medial meniscus surface contacting tibial cartilage - *mmtib*
- medial meniscus surface contacting femoral cartilage - *mmfem*

Depending on the pre-processor, these set definitions will be available once the mesh file is imported. For example, PreView, the pre-processor for FEBio, converts all the set definitions to groups. Element, node, and surface sets were simply defined to aid in model generation by easing assignment of materials, interactions, loads, and boundary conditions.

Tools

The distribution includes a variety of scripts to facilitate mesh generation and automate development of the model from a mesh file. Advanced users may want to work with these scripts for customization of the model.

Mesh Generation

Mesh generation was done using TrueGrid (XYZ Scientific, Livermore, CA). It is possible to generate high quality hexahedral meshes by using surface projection methods in this software, through a graphical interface or in a scripted fashion. Describing how to use TrueGrid is beyond the scope of this document. Nonetheless, the script used to generate the knee mesh is provided in the following location:

<PATH_TO_UNCOMPRESSED_DISTRIBUTION>/src/tf_joint.tg

The script utilizes the geometric models described in the Geometry section above and generates the mesh file (see Mesh section above), including all the node, element, and surface sets. Note that, the mesh script is compatible with TrueGrid 2.2.6 but NOT later versions i.e. 2.3.4.

Model Generation

A Python script (minimum version 2.6.5) utilizing the SciPy (http://www.scipy.org) library is used to automate model generation in FEBio XML format. While the following information is provided in the Quick Start section, it was repeated here for convenience. Model generation with scripting relies on two main files (as noted below) and also the mesh file noted in the configuration file (see details below):

<PATH_TO_UNCOMPRESSED_DISTRIBUTION>/src/abq2feb.py

<PATH_TO_UNCOMPRESSED_DISTRIBUTION>/src/abq2feb.cnfg

Changing the model using this approach involves updating the configuration file (abq2feb.cnfg), executing the Python script (abq2feb.py) with this configuration, which in turn generates an FEBio file for the updated model:

- *Configuration* (abq2feb.cnfg) – Configuration file includes specifications of many model attributes: output model name, path of the mesh file (text based Abaqus input file format, *.inp), solver/time settings, material properties, interactions, loads, boundary conditions, and anatomical locations for transformation to a physiological coordinate system. Execution of the python script with the supplied configuration file will generate the

distributed model file, tf_joint.feb, by overwriting it. Keywords in the configuration file are identified by '#' and comments by '##'. It should be noted that node, element, and surface sets referenced in the configuration file are case-sensitive and must be referenced exactly as in the mesh file.

- *Execution* (abq2feb.py) – To execute the script, the user needs to navigate to the directory containing the script and the configuration file (by default abq2feb.cnfg) in the command prompt. In following, execution of the following command will generate a model file using the output model name defined in the configuration file:

  python abq2feb.py -- abq2feb.cnfg

  One can substitute the configuration file. In Linux systems, it may be necessary to omit the '--'.
- *Output* (*.feb) – Execution of the Python script results in an FEBio model file reflecting the content of the configuration file. The filename will be based on the model entry in the configuration file. By default, the distribution results in tf_joint.feb in the same folder as the script and the configuration file.

Model

The following information is specific to the model file provided in the release package. The user can change any of these properties/definitions to fit his/her needs. This model relies on the imaging data, geometric models, and mesh described previously (see Data section) and generated using the mesh and model generation scripts (see Tools section) (Figure 4). The model is defined in FEBio (http://mrl.sci.utah.edu/software/febio) syntax and can be found at the following location:

<PATH_TO_UNCOMPRESSED_DISTRIBUTION>/src/tf_joint.feb

Geometry & Mesh

The geometry and mesh used in the model are described in the Data section. To accommodate an anatomically based coordinate system, nodes of the mesh were transformed in a way that the model coordinate system is consistent with a widely accepted coordinate system used to describe tibiofemoral joint movements [2] (Figure 5). For this purpose, coordinates of the following points were extracted on the original mesh:

- medial femoral condyle - *medial_f_cond*
- lateral femoral condyle - *lateral_f_cond*
- proximal femur - *proximal_femur*
- distal femur - *distal_femur*
- proximal tibia – *proximal_tibia*

An approximate Q-angle of 14.1° (measured as angle between distal to proximal femur vector and superior direction axis) and was defined for the specimen [3]. This angle was achieved by prescribing a 5.3° rotation in the frontal plane to the knee in original coordinate system since the initial Q-angle was 8.8°. Mechanical axis of the femur was calculated by rotating the unit vector

from distal femur to proximal femur by the Q-angle. A temporary mediolateral axis was defined by the unit vector from medial condyle to lateral condyle. An anterior-posterior axis was defined by the cross product of the mechanical axis and the mediolateral axis. The flexion axis was defined by the cross product of the anterior-posterior axis and the mechanical axis. Using this information, the new model coordinate system was defined as:

- origin at the mid-point of medial and femoral condyles
- x-axis from the flexion axis
- y-axis from the anterior-posterior axis
- z-axis from the mechanical axis

This process was conducted by using the script for model generation (see Tools section). It should be noted that while the whole model was transformed into an anatomical coordinate system, prescription of kinematics exactly based on the Grood and Suntay system [2] is still not possible at this moment. All rotations and translations are given in the model coordinate system. While flexion can be described by rotation about the approximate flexion axis in the current implementation, prescription of movements of the other axes require more elaborate definition of multiple coordinate systems, i.e. floating axis, or a pre-processing effort.

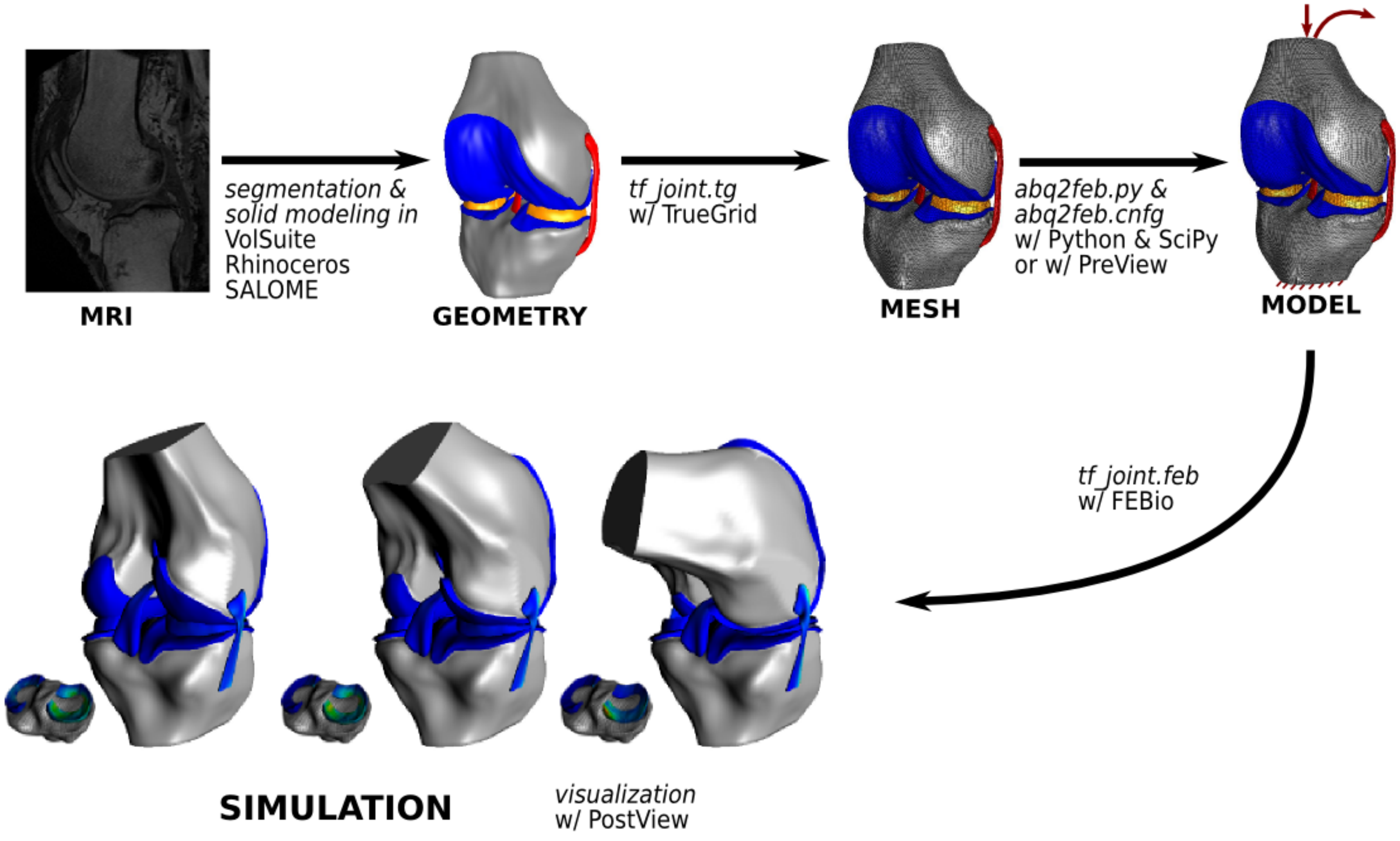

Figure 4. Modeling pipeline.

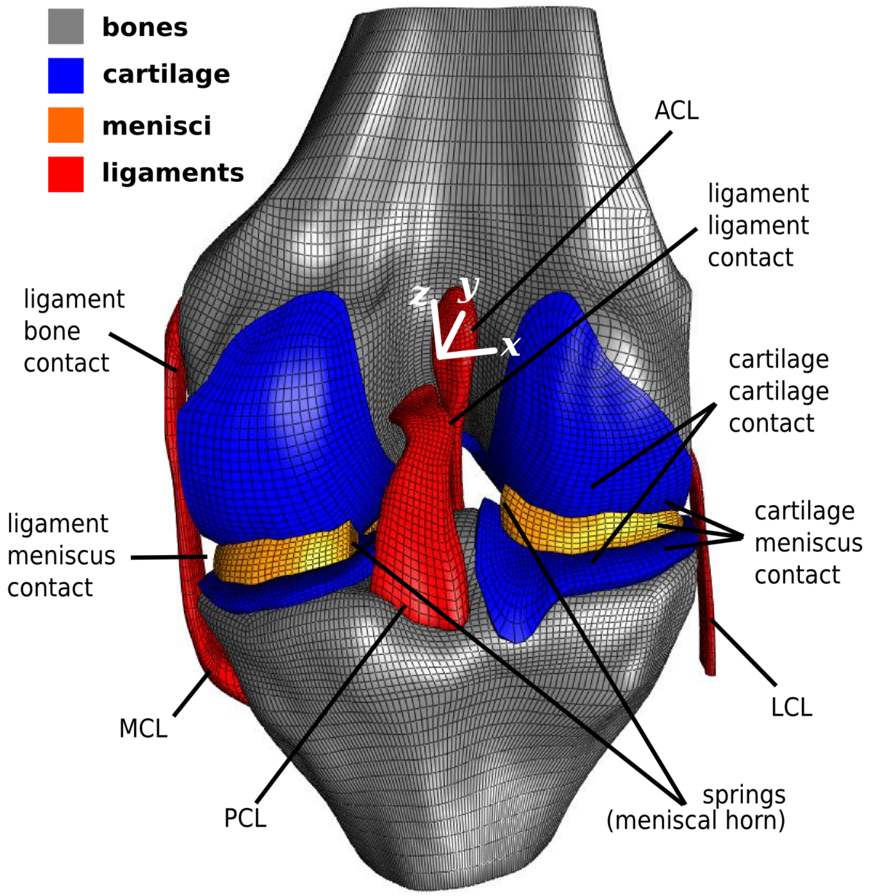

Figure 5. An overview of model components; not all model details are illustrated. Bones were assumed to be rigid and all other tissue structures were modeled as nonlinearly elastic. For femur and tibia, rigid body reference points were coincident with the origin of the model coordinate system. All loading and boundary conditions for the bones, e.g. tibiofemoral joint loading and kinematics, were defined at these points in model coordinate system.

Material Properties

Constitutive models available in FEBio were utilized to define material behavior. All material properties were based on values reported in literature, the parameters are provided in self-consistent units such that derived units can be expressed in terms of fundamental units.

Bones

Table 3. Properties used for the bones [4].

**Bone**	**Density (tonnes/mm^3)**
Femur	1.132e-6
Tibia	1.132e-6

For cost-effective simulations and due to large stiffness of bones relative to soft tissue structure, femur and tibia were defined as rigid bodies. This greatly reduces the number of equations in the system and allows for motion to be defined through rigid body kinematics. Bone densities were assigned to approximate 1000 times mass scaled value for dry cortical bone void of

cancellous (Table 3). In addition to mass scaling, it should also be noted that the overall mass and inertia of the bones are not accurate due to description of bone surfaces only using shells. Nonetheless, the inertial properties are not critical for quasi-static simulations or slow dynamic simulations.

Ligaments

Ligaments were defined as nearly incompressible, transversely isotropic hyperelastic fiber material [5] with a Mooney-Rivlin [6] ground substance as defined by the following strain energy function:

$$W = C_1(\tilde{I}_1 - 3) + C_2(\tilde{I}_2 - 3) + \frac{K}{2}(\ln(J))^2 + F(\tilde{\lambda}) \tag{1}$$

with

$$\tilde{\lambda}\frac{\partial F}{\partial \tilde{\lambda}} = \begin{cases} 0 & \tilde{\lambda} < 1 \\ C_3(e^{C_4(\tilde{\lambda}-1)} - 1) & 1 \leq \tilde{\lambda} < \lambda_m \\ C_5 + C_6\tilde{\lambda} & \tilde{\lambda} \geq \lambda_m \end{cases} \tag{2}$$

where,

C_1 and C_2: Mooney-Rivlin constants (MPa)

$C_3 - C_6$: Fiber material function constants (C_3, C_5, and C_6 in MPa; C_4 unitless)

K: Bulk modulus like penalty parameter (MPa)

$\tilde{I}_1$: first invariant of the deviatoric right Cauchy-Green deformation tensor

$\tilde{I}_2$: second invariant of the deviatoric right Cauchy-Green deformation tensor

J: determinant of the deformation gradient tensor (volume ratio)

F: strain energy density component based on the deviatoric fiber stretch

$\tilde{\lambda}$: Deviatoric part of the stretch along fiber direction

λ_m : Straightened fiber stretch

The parameters for the representation of ligament material response were adapted from literature and reported in Table 4.

Table 4. Properties used for the ligaments. Density units are in tonnes/mm^3. When applicable material coefficient units are in MPa.

Ligament	Density	C_1	C_2*	K	C_3	C_4	C_5	λ_m	Source
ACL	1.5e-9	1.95	0	73.2	0.0139	116.22	535.039	1.046	[7]
PCL	1.5e-9	3.25	0	122	0.1196	87.178	431.063	1.035	[7]
MCL	1.5e-9	1.44	0	397	0.57	48	467.1	1.063	[8]
LCL	1.5e-9	1.44	0	397	0.57	48	467.1	1.063	[8]

*C_2 is set to zero to reduce the ground substance to a Neo-Hookean material [6].

Cartilage

Currently, all three layers of cartilage were defined as a nearly incompressible Mooney-Rivlin material, with the strain energy function given as:

$$W = C_1(\tilde{I}_1 - 3) + C_2(\tilde{I}_2 - 3) + \frac{K}{2}(\ln(J))^2 \qquad (3)$$

where,

C_1 and C_2: Mooney-Rivlin constants (MPa)

K: Bulk modulus like penalty parameter (MPa)

$\tilde{I}_1$: first invariant of the deviatoric right Cauchy-Green deformation tensor

$\tilde{I}_2$: second invariant of the deviatoric right Cauchy-Green deformation tensor

J: determinant of the deformation gradient tensor (volume ratio)

The parameters for the representation of cartilage material response were adapted from literature and reported in Table 5. This representation of the cartilage will likely be changed in the future with the implementation of a more physiological constitutive models.

Table 5. Properties used for the cartilage. Density units are in tonnes/mm^3. When applicable material coefficient units are in MPa.

Density	C_1	C_2*	K+	Source
1.5e-9	0.856	0	8	[9]

*C_2 is set to zero to reduce the ground substance to a Neo-Hookean material [6].

+K set to correspond to approximately 0.46 Poisson ratio.

Menisci

The menisci were defined as a Fung orthotropic hyperelastic material, with the strain energy function given as:

$$W = \frac{1}{2}c(e^Q - 1)$$

$$Q = c^{-1}\sum_{a=1}^{3}[2\mu_a \boldsymbol{A}_a^0 : \boldsymbol{E}^2 + \sum_{b=1}^{3}\lambda_{ab}(\boldsymbol{A}_b^0 : \boldsymbol{E})]$$

with

$$\begin{bmatrix} \lambda_{11}+2\mu_1 & \lambda_{12} & \lambda_{13} \\ \lambda_{12} & \lambda_{22}+2\mu_2 & \lambda_{23} \\ \lambda_{13} & \lambda_{23} & \lambda_{33}+2\mu_3 \end{bmatrix} = \begin{bmatrix} \frac{1}{E_1} & \frac{-\nu_{12}}{E_1} & \frac{-\nu_{31}}{E_3} \\ \frac{-\nu_{12}}{E_1} & \frac{1}{E_2} & \frac{-\nu_{23}}{E_2} \\ \frac{-\nu_{31}}{E_3} & \frac{-\nu_{23}}{E_2} & \frac{1}{E_3} \end{bmatrix} \qquad (4)$$

$$\mu_1 = G_{12} + G_{31} - G_{23}$$
$$\mu_2 = G_{12} - G_{31} + G_{23}$$
$$\mu_3 = -G_{12} + G_{31} + G_{23}$$

where,

c: N/A

$\boldsymbol{A}^0$: relates to initial direction of material axes

$\boldsymbol{E}$: Green-Lagrange strain tensor

λ : Lamé's first parameter

μ : Lamé's second parameter

E: Young's modulus (MPa)

G: Shear modulus (MPa)

v: Poisson's ratio

K: Bulk modulus like penalty parameter (MPa)

The parameters for the representation of menisci material response were adapted from literature and reported in Table 6.

Table 6. Properties used for the menisci. Density units are in tonnes/mm^3. When applicable material coefficient units are in MPa.

Density	E_1	E_2	E_3	ν_{12}	ν_{23}	ν_{31}	G_{12}	G_{23}	G_{31}	c*	K*	Source
1.5e-9	125.0	27.5	27.5	0.1	0.33	0.1	2.0	12.5	2.0	1.0	10.0	[10]

* Currently, K and c have no effect on material behavior. They are set for FEBio syntax compliance [11].

When necessary, constants were converted to shear and bulk moduli to make use of FEBio's decoupled hyperelastic materials. This was done using the following relationships:

$$G=\frac{E}{2(1+v)} \tag{5}$$

$$K=\frac{E}{3(1-2v)} \tag{6}$$

Interactions

Tissue components in the knee joint are likely to have mechanical interactions in between. Such interactions include attachment of soft tissues to the bones, e.g. ligament insertion sites, and contact between tissue segments as the loads and boundary conditions are applied to the tibiofemoral joint.

Rigid Interfaces

As bones were defined to be rigid, one way to represent the attachment of cartilage and ligaments to the bones relies on definition of rigid interfaces. This way, the selected nodes of the soft tissue will be constrained to move with the rigid body they are assigned to. Four such interfaces are defined in the Open Knee model:

- Femoral cartilage to femur - *cart2fem*
- Tibial cartilage (medial and lateral) to tibia - *cart2tib*
- Ligament origins (both cruciate and collateral ligaments) to femur - *lig2fem*
- Ligament insertions (both cruciate and collateral ligaments) to tibia - *lig2tib*

Contact

Contact between bones and tissues, in between articulating surfaces, as well as between ligaments were defined based on the finite sliding contact formulation in FEBio (Figure 5). Contact is frictionless; it relies on a two-pass facet-to-facet contact algorithm to negate the effects

of potential mesh density mismatches. Calculation of contact variables relies on penalty stiffnesses formulation of FEBio [6], with the stiffness prescribed as 100 MPa/mm This approach was determined to yield convergent contact behavior in this problem therefore it is used for all contact definitions. The following contact interfaces are defined in FEBio to accommodate physiological mechanical interactions:

- medial femoral cartilage to medial tibial cartilage - *mcart2mcart*
- lateral femoral cartilage to lateral tibial cartilage - *lcart2lcart*
- medial femoral cartilage to medial meniscus - *fcart2mmeni*
- lateral femoral cartilage to lateral meniscus - *fcart2lmeni*
- medical tibial cartilage to medial meniscus - *tcart2mmeni*
- lateral tibial cartilage to lateral meniscus - *tcart2lmeni*
- femur to medial collateral ligament – *femur2mcl* (inactive)
- tibia to medial collateral ligament – *tibia2mcl* (inactive)
- femur to lateral collateral ligament - *femur2lcl* (inactive)
- tibia to lateral collateral ligament - *tibia2lcl* (inactive)
- anterior cruciate ligament to posterior cruciate ligament - *acl2pcl*

Other Components

Meniscal horn attachments were defined using linear springs attaching each node on the meniscal horn faces to a node on the tibia approximately intersecting the normal of the horn face extended from the approximate face centroid (Figure 5). The spring constants were calculated from reported Young's modulus for the horn attachments [12]:

$$k_i = \frac{E}{NL_i} A \qquad (7)$$

where, k_i is the ith spring stiffness, L_i is the ith spring length, E is the Young's modulus of the meniscal horn, A is the total horn face area, and N is the number of nodes on that face. The spring length for each spring was calculated from the insertions of the spring at the node of the horn face and at the tibial node. Horn specific parameters are provided in Table 7. Note that in the current FEBio implementation (version 1.3.0), these springs are both for compression and tension.

Table 7. Parameters used to represent mechanical properties of the meniscal horn attachments [12].

**Meniscal Horn**	***E* (MPa)**	***A* (mm^2)**	***N***
Lateral-anterior	600	37.2	88
Lateral-posterior	600	24.58	88
Medial-anterior	600	37.2	88
Medial-posterior	600	24.58	88

Loading & Boundary Conditions

The model was setup in a way that it can simulate passive flexion. The tibia is fixed in all six degrees of freedom (DoF) while the femur is free. Passive flexion is defined by first applying a desired compressive force (in N) along the z-axis (proximal-distal) to the femur at the joint center through a linear ramp function to a desired time point. During this time, the flexion angle is held at 0°. In the remainder of the simulation, the compressive force is held constant and a desired flexion angle (in radians) can be applied to the femur about the x-axis (medial-lateral) as a linear ramp function. The 'load curve' definitions in FEBio file (tf_joint.feb) and model generation script (abq2feb.cnfg) can be edited to reflect different values for loading and flexion angle as well as can be adopted to incorporate different loading modes.

Output

By default, FEBio stores a variety of displacement, strain, and stress variables in a binary file, *plotfile* (*.plt) (see FEBio manual for details [11]). This file is generated after running a simulation (see Quick Start section). In addition to the standard outputs, contact pressures at all contact interfaces (see Interactions section) are also requested to be written to the plot file. With the current output settings for the Open Knee model, the outputs are written to the plotfile at time increments of 0.1 seconds. It is possible to view the contents of the file in PostView (http://mrl.sci.utah.edu/software/postview), the post-processing software for FEBio.

Simulations with FEBio also generates a *logfile* (*.log), a text file, which provides the same output as the screen while FEBio runs. The logfile contains detailed information on convergence of simulation, e.g. summary of iterations. In the Open Knee model additional data is requested to be written in this logfile, in particular rigid body kinematics and kinetics for femur and tibia:

- x-coordinate of center of mass (x)
- y-coordinate of center of mass (y)
- z-coordinate of center of mass (z)
- x-component of rotation quaternion (qx)
- y-component of rotation quaternion (qy)
- z-component of rotation quaternion (qz)
- w-component of rotation quaternion (qw)
- x-component of rigid body reaction force (Fx)
- y-component of rigid body reaction force (Fy)
- z-component of rigid body reaction force (Fz)
- x-component of rigid body reaction torque (Mx)
- y-component of rigid body reaction torque (My)
- z-component of rigid body reaction torque (Mz)

These data are written to the logfile for each converged increment of the simulation. All these variables can be used to calculate relative tibiofemoral joint kinematics and kinetics in any

desired kinematic convention, e.g. that of [1].

Case Simulations

A case simulation was conducted using the distributed Open Knee model (see Model section), with case-specific loading and boundary conditions.

Table 8. Simulation settings. For more details on these parameters, please refer to FEBio manual [6], [11].

Setting	Value	Description
title	tf_joint	Title of the problem
time_steps	25	Total number of time steps
step_size	0.1	Initial time step size
max_refs	100	Maximum number of stiffness reformations
max_ups	0	Maximum number of BFGS stiffness updates; the provided settings invokes full-Newton iteration
dtol	0.01	Convergence tolerance on displacements
etol	0.01	Convergence tolerance on energy
rtol	0.1	Convergence tolerance on residual
lstol	0.5	Convergence tolerance on line search
pressure_stiffness	0	Flag to use the surface pressure stiffness term in the calculation of the global stiffness matrix
aggressiveness	1	Time-step cutback algorithm; the provided setting allows cutting back the time step size by half if convergence is not likely
dtmin	0.001	Minimum time step size
dtmax	lc=”1”	Maximum time step size; the provided setting dictates maximum time step using the load curve with the id “1”
max_retries	10	Maximum number of time step cutbacks allowed
opt_iter	10	Optimal number of iterations
plot_level	PLOT_MUST_POINTS	Solution increments to be saved in the plotfile; the provided settings invokes saving of the solution at the must points, i.e. based on the load curve provided for dtmax
analysis	dynamic	Analysis type; the provided setting invokes implicit dynamic analysis

Solver Software

FEBio was employed for all finite element analysis. It is a freely available, open-source, non-linear finite element analysis package tailored to solve biomechanics problems. The software can be downloaded from http://mrl.sci.utah.edu/software/febio. Additional information is also available at the same site. The distributed model and simulation scenarios were tested using

FEBio version 1.3.0.1428. For more details on running simulations using FEBio, please refer to Quick Start section.

Simulation Settings

Simulation settings dictate control parameters to conduct finite element analysis, in particular the analysis type, parameters for iterations and convergence tolerances. These are specified in the distributed model file (tf_joint.feb); can be adjusted directly by editing that file, or by the provided model generation script (see Tools section). For more details on implications of these settings, the users are advised to refer to FEBio manual [6], [11]. Settings used in the case simulations are summarized in Table 8. The linear solver was set to “pardiso” to benefit the multi-threading capabilities [13], [14].

Case 1: Passive Knee Flexion

The model, *tf_joint.feb*, was used to simulate passive flexion under a low constant compressive load. The simulation occurred over a 2.5 second time interval with loads and boundary conditions described in Table 9.

Table 9. Loading scenario used for simulating passive knee flexion.

	**Femur**					
**Time Interval**	**x**	**y**	**z**	θ_x	θ_y	θ_z
0 - 1 second	free	free	-100 N ramp	0	free	free
1 - 2.5 second	free	free	-100 N constant	0.785 radian ramp	free	free
	**Tibia**					
**Time Interval**	**x**	**y**	**z**	θ_x	θ_y	θ_z
0 - 1 second	fixed	fixed	fixed	fixed	fixed	fixed
1 - 2.5 second	fixed	fixed	fixed	fixed	fixed	fixed

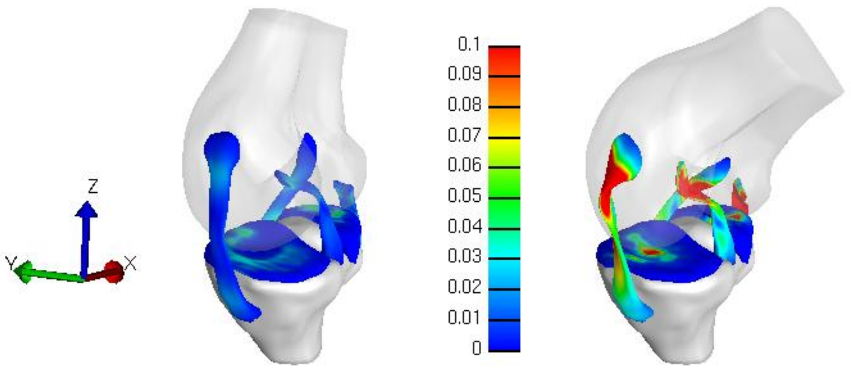

Figure 6. Effective Green-Lagrange tissue strain during passive knee flexion. The left image shows effective strain resulting from 100 N compressive force prior to rotation. The right image shows effective strain at 45 degree flexion (0.785 radians), 100 N compressive load. Menisci and femoral cartilage are hidden for purpose of visualization.

The wall clock time for this simulation was approximately 12 hours (using 8 threads on a 24 GB RAM, dual socket quad core 2.5 GHz AMD Opteron Linux computing node at Ohio Supercomputer Center). Strain distribution in soft tissues can be seen in Figure 6; the predicted tibiofemoral axial rotation and varus/valgus kinematics (in Grood and Suntay coordinate system) are illustrated in Figure 7 It should be noted that this simulation is predictive in nature since all non-prescribed femoral degrees of freedom are free to change as a result of loading. While this specific simulation scenario does not have a corresponding experimental dataset, the procedure to conduct this simulation can be utilized to impose experimentally available loading and kinematics cases. For example, experimentation was conducted for combined loading in which anterior-posterior, varus-valgus, and internal-external forces and moments were applied with the flexion angle fixed and all other degrees of freedom free (flexion was fixed) [1]. This model can later be employed with boundary conditions and loads equivalent to such experimental values to assess its specimen-specific predictive capabilities, as well as sensitivity to constitutive model, mesh density, and coordinate system accuracy.

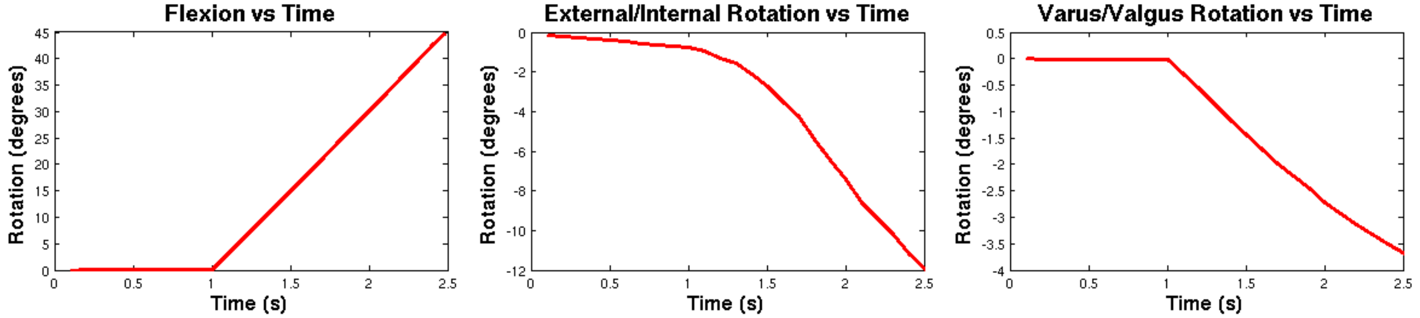

Figure 7. Tibiofemoral joint rotation kinematics predicted for passive knee flexion (tibia-relative to femur).

Verification and Validation

Models used in finite element analysis need to be supported by verification and validation studies to establish confidence in simulation predictions [15]. Open Knee is not an exception. Nonetheless, this version was focused towards early dissemination, providing the opportunity for others to conduct extensive simulations using the model to explore problem-specific sensitivity and validity. In this section, we'll discuss potential directions for verification and validation, particularly for our desired utility of the model.

Solver Verification

Open Knee relies on FEBio for simulation of knee biomechanics. Verification of numerical procedures and simulation approaches employed by FEBio is provided through a verification suite, which can be downloaded at the software site (http://mrl.sci.utah.edu/software/febio). The users of Open Knee are encouraged to explore these test problems and consult FEBio related publications [16] to establish confidence in this software package.

Sensitivity to Simulation Settings

Simulations parameters for the Open Knee model were set to conduct an implicit dynamic

analysis [17]. In this case, the inertial response of the system was modeled and the time histories of loading and boundary conditions is not abstract, as in implicit static analysis. At each time increment, the analysis procedure seeks to solve nonlinear sets of equations via Newton's method [6], which is different than the well-known explicit finite element analysis [18] which simply needs to perform forward integration, but is subject to time step size instability. It is possible for users to switch to quasi-Newton method to decrease solution time yet with the disadvantage of approximation of the stiffness matrix. The convergence tolerances for the solution of system equations can also be changed. Different analysis types, e.g. quasi-static, can also be explored. Nonetheless, one should note that finite element analysis of the knee, as described in here, is highly nonlinear and changing these simulation settings do not guarantee that a solution can be obtained. The influence of different simulation settings on model predictions is yet to be documented. All those settings can be changed by the provided model generation script and configuration file (see Model Generation component of Tools section), or directly in the FEBio model (see Model section). The users are encouraged to refer to FEBio manual on the implications of these simulation settings (see FEBio site at http://mrl.sci.utah.edu/software/febio).

Mesh Convergence

Mesh density of the Open Knee is comparable to other published knee models [9], [7], [10]. Mesh generation for thin structures, e.g. cartilage, was conducted in a way that there were at least three elements along the thickness of the tissue. However, prospective mesh convergence analysis is still warranted to confirm that model predictions are not a function of mesh density. Given the structured mesh generation process and automated model development (see Tools section), the mesh density can be changed easily (for the whole model or for individual components) and model can be rebuilt. It should be noted that a satisfactory mesh density to reproduce joint response may not be sufficient for the analysis of tissue stresses, e.g. cartilage contact pressures. Therefore tissue-specific mesh convergence analysis may be necessary depending on the desired utility of the model.

Repeatability & Reproducibility

As in any finite element analysis study, the predictions of the Open Knee model should be repeatable and reproducible. Given FEBio, the finite element analysis package used to solve the disseminated model, simulations conducted on different hardware platforms should provide the same results. To allow users to run the model on their platforms and compare their predictions against ours, results of a sample loading scenario was also included in the package (see Case Simulations section). The users are advised to contact the Open Knee development team and the developers of FEBio ((http://mrl.sci.utah.edu/software/febio) to report any discrepancies.

The users are also encouraged to rebuilt the model from scratch, starting with the mesh file, to test if they can reproduce model development and simulation stages using FEBio. Alternatively, they can reproduce the model in another finite element analysis software package, e.g. Abaqus (Simulia, Providence, RI), to compare findings. One should note that defining the model in the same way may not be possible due to differences in built-in capabilities of solvers but equivalent

representations may be possible, particularly for the constitutive models.

Validation

The users should consider validation of the knee model based on the requirements of the targeted utility of the model and the variables of interests. Validation studies therefore can be done to evaluate the mechanical response of the whole joint or tissue level stress-strain response. The specimen used in the Open Knee has a wide range of mechanical testing data conducted on the intact joint and after the dissection of anterior cruciate ligament [1]. In the upcoming versions of the Open Knee, our goal is to conduct direct validation of the mechanical response of the joint based on simulations reproducing these experimental joint loading conditions. Currently, validation of the Open Knee is minimal; only qualitatively based on general trends for the simulated loading situations, also observed in literature (see Case Simulations section).

Validity of the Open Knee to predict tissue stresses in physiological knee joint loading is yet questionable. Unfortunately, specimen-specific measurements on the tissues are not available. Therefore, any assessment of tissue deformation predictions should rely on indirect validation based on comparisons against previous studies. For example, contact pressures between articulating surfaces can be evaluated against other simulation results available in literature [9], [7], [10], after simulating the Open Knee model under the same loading conditions. Similarly, consistency of predictions of internal tissue deformations may be established based on previous studies [10]. One should note that tissue related subsidiary models, i.e. material models, are based on literature and their suitability for this specific specimen is not established. Nonetheless, it may be possible to use inverse finite element analysis [19] to adjust these parameters such that a subset of joint level data is reproduced adequately [1]. The rest of the data can be used to explore the validity of these adjusted parameters.

Sensitivity to Model Parameters

While joint level validation can be established in future, due to lack of specimen-specific experimental data on tissue level response, e.g. for tissue strains under physiological loading, it may not be possible to conduct direct validation. Nonetheless, the users are advised to explore sensitivity of model outputs on model parameters that have uncertainties. Similar to prospective validation studies, the users should consider sensitivity analysis based on the requirements of the targeted utility of the model and the variables of interests. Both joint level and tissue level responses may be affected by uncertainties in the selected material properties for tissues, geometric inaccuracies, errors in loading and boundary conditions, and modeling assumptions in various other model components, e.g. meniscal attachments. As of this version, the sensitivity map of the Open Knee model on joint and tissue parameters has not been reconstructed.

Known Limitations

As in many modeling efforts, finite element representation of the knee in the Open Knee project also has inherent limitations. These may have significant influence on the model's predictive capacity depending on the purpose of its utilization. Here we provide a classified list of limitations that we are aware of. It should be noted that in future, some of these issues can be

addressed by the Open Knee team or by others who have access to raw data utilized for model development.

- Joint Representation
  - Knee joint experimentation, including robotics testing for mechanical characterization of the joint and imaging, was conducted on the intact knee. The current model on the other hand do not have representations of patella, patellar tendon, joint capsule, skin, etc.
  - Attachment of medical collateral ligament to medial meniscus is not modeled.
  - Attachment of medial meniscus periphery to tibial cartilage is not modeled.
- Anatomical Reconstruction
  - Geometries of knee structures were obtained through manual segmentation, which may induce inaccuracies in surface representations.
  - Due to low contrast of magnetic resonance imaging for meniscus reconstruction, the geometry for lateral and medial menisci insertion regions are incomplete, potentially indicating inaccurate representation of insertion locations.
  - Due to low resolution of magnetic resonance imaging for lateral collateral ligament reconstruction, lateral collateral ligament geometry was approximated with its fibular insertion zone partially modeled.
  - Due to low resolution of magnetic resonance imaging for medial collateral ligament reconstruction, the geometry of this ligament may have inaccuracies.
- Constitutive Models
  - Material properties of tissues are entirely based on literature, they are not specimen-specific.
  - Ligament pre-strains are not defined, the stress-strain distributions within the ligaments will likely be sensitive to this component.
  - Anisotropy of the cartilage is not modeled, the stress-strain distribution within the cartilage should be evaluated in view of this information.
  - Bone densities are mass scaled to facilitate dynamic simulations, influence of inertial effects on simulation results may need to be quantified.
- Verification & Validation
  - At this moment, the validation can only rely on qualitative evaluation of joint response against literature data. As a result, tissue stress-strain distributions should be considered with a grain of salt.
  - Determination of mesh density relied on visual inspection and to ensure at least three elements along the thickness of tissues, e.g. cartilage and ligaments. Nonetheless, elaborate mesh convergence studies are needed to ensure that study-dependent predictions are not influenced by selection of mesh density.

Citing Open Knee

The model distributed by this project can be cited by referring to this guide, the website, or the literature utilizing the model. Please include one or more of the following to cite this work:

- Erdemir A. and Sibole S. *Open Knee: A Three-Dimensional Finite Element Representation of the Knee Joint, User's Guide, Version 1.0.0*, December 17, 2010.
- *Simtk.org: Open Knee: A Three-Dimensional Finite Element Representation of the Knee Joint*, https://simtk.org/home/openknee.
- Sibole, S., Bennetts, C., Borotikar, B., Maas, S., van den Bogert, A. J., Weiss, J. A. and Erdemir, A. *Open knee: a 3D finite element representation of the knee joint*, 34th Annual Meeting of the American Society of Biomechanics, August 18-21, 2010, Providence, RI.

Access and Contact Information*

Website

https://simtk.org/home/openknee

Wiki

http://wiki.simtk.org/openknee (bleeding edge documentation)

Repository

https://simtk.org/svn/openknee (bleeding edge source code; a Subversion client is helpful to checkout the full source code)

Forums

https://simtk.org/forum/?group_id=485 (users' and developers' discussion)

Correspondence

Ahmet Erdemir, PhD
Department of Biomedical Engineering, ND20
Lerner Research Institute, Cleveland Clinic
9500 Euclid Avenue, Cleveland, OH 44195, USA
phone: +1 (216) 445 9523
fax: +1 (216) 444 9198
e-mail: erdemira@ccf.org

Scott Sibole, BSc
Department of Biomedical Engineering, ND20
Lerner Research Institute, Cleveland Clinic

9500 Euclid Avenue, Cleveland, OH 44195, USA
phone: +1 (216) 444 1902
fax: +1 (216) 444 9198
e-mail: siboles@ccf.org

*Valid as of December 17, 2010.

Acknowledgments

Model development efforts were supported by National Institute of Biomedical Imaging and Bioengineering, National Institutes of Health (R01EB009643; Erdemir, Principal Investigator). Data collection was supported by National Institute of Arthritis and Musculoskeletal and Skin Diseases, National Institutes of Health (R01AR049735; Hewett, Principal Investigator & van den Bogert, Co-Investigator); the procedures were part of the doctoral work conducted by Bhushan Borotikar. FEBio, finite element analysis software, was supported by National Institute of General Medical Sciences, National Institutes of Health (R01GM083925; Weiss & Ateshian, Principal Investigators). Hosting for the project is provided by SimTK (https://simtk.org). SimTK, the Simulation Toolkit, is a part of Simbios project funded by the National Institutes of Health through the NIH Roadmap for Medical Research (U54GM072970; Altman, Principal Investigator). Access to high performance computing was provided by Ohio Supercomputer Center (http://www.osc.edu).

Efforts by freely available and open source software development teams, in particular FEBio (http://mrl.sci.utah.edu/software/febio), VolSuite (http://www.osc.edu/archive/VolSuite) and SALOME (http://www.salome-platform.org), are highly praised. The development team also appreciates academic/research discounts provided by software manufacturers TrueGrid (XYZ Scientific Applications, Inc., Livermore, CA, USA) and Rhinoceros (McNeel North America, Seattle, WA, USA).

References

[1] B. Borotikar, “Subject specific computational models of the knee to predict anterior cruciate ligament injury, Doctoral Dissertation,” *Cleveland State University*, Dec. 2009.

[2] E. S. Grood and W. J. Suntay, “A joint coordinate system for the clinical description of three-dimensional motions: application to the knee,” *Journal of Biomechanical Engineering*, vol. 105, no. 2, pp. 136-144, May. 1983.

[3] L. Herrington and C. Nester, “Q-angle undervalued? The relationship between Q-angle and medio-lateral position of the patella,” *Clinical Biomechanics (Bristol, Avon)*, vol. 19, no. 10, pp. 1070-1073, Dec. 2004.

[4] K. W. K. Yuen et al., “Characteristics of age-related changes in bone compared between male and female reference Chinese populations in Hong Kong: a pQCT study,” *Journal of Bone and Mineral Metabolism*, 2010.

[5] J. C. Gardiner, J. A. Weiss, and T. D. Rosenberg, “Strain in the human medial collateral ligament during valgus loading of the knee,” *Clinical Orthopaedics and Related Research*, no. 391, pp. 266-274, Oct. 2001.

[6] S. Maas, D. Rawlins, J. Weiss, and G. Ateshian, “FEBio Finite Elements for Biomechanics: Theory Manual,” Jul-2010.

[7] E. Peña, B. Calvo, M. A. Martínez, and M. Doblaré, “A three-dimensional finite element analysis of the combined behavior of ligaments and menisci in the healthy human knee joint,” *Journal of Biomechanics*, vol. 39, no. 9, pp. 1686-1701, 2006.

[8] J. C. Gardiner and J. A. Weiss, “Subject-specific finite element analysis of the human medial collateral ligament during valgus knee loading,” *Journal of Orthopaedic Research: Official Publication of the Orthopaedic Research Society*, vol. 21, no. 6, pp. 1098-1106, Nov. 2003.

[9] T. L. H. Donahue, M. L. Hull, M. M. Rashid, and C. R. Jacobs, “A finite element model of the human knee joint for the study of tibio-femoral contact,” *Journal of Biomechanical Engineering*, vol. 124, no. 3, pp. 273-280, Jun. 2002.

[10] J. Yao, J. Snibbe, M. Maloney, and A. L. Lerner, “Stresses and strains in the medial meniscus of an ACL deficient knee under anterior loading: a finite element analysis with image-based experimental validation,” *Journal of Biomechanical Engineering*, vol. 128, no. 1, pp. 135-141, Feb. 2006.

[11] S. Maas, D. Rawlins, J. Weiss, and G. Ateshian, “FEBio Finite Elements for Biomechanics: User's Manual,” Jun-2010.

[12] K. N. Hauch, M. L. Oyen, G. M. Odegard, and T. L. Haut Donahue, “Nanoindentation of the insertional zones of human meniscal attachments into underlying bone,” *Journal of the Mechanical Behavior of Biomedical Materials*, vol. 2, no. 4, pp. 339-347, Aug. 2009.

[13] O. Schenk and K. Gärtner, “On fast factorization pivoting methods for symmetric indefinite systems,” *Elec. Trans. Numer. Anal.*, vol. 23, pp. 158-179, 2006.

[14] O. Schenk and K. Gärtner, “Solving Unsymmetric Sparse Systems of Linear Equations with PARDISO,” *Journal of Future Generation Computer Systems*, vol. 20, no. 3, pp. 475-487, 2004.

[15] A. E. Anderson, B. J. Ellis, S. A. Maas, C. L. Peters, and J. A. Weiss, “Validation of finite element predictions of cartilage contact pressure in the human hip joint,” *Journal of Biomechanical Engineering*, vol. 130, no. 5, p. 051008, Oct. 2008.

[16] S. A. Maas, B. J. Ellis, D. S. Rawlins, and J. A. Weiss, “A Comparison of FEBio, ABAQUS, and NIKE3D Results for a Suite of Verification Problems,” *SCI Technical Report, No. UUSCI-2009-009, SCI Institute, University of Utah*, 2009.

[17] N. M. Newmark, “A Method of Computation for Structural Dynamics,” *Journal of Engineering*

Mechanics Division ASCE, vol. 85, no. 3, pp. 67-94, 1959.

[18] K. Bathe, “Solution of Nonlinear Equations in Dynamic Analysis,” in *Finite Element Procedures*, Prentice-Hall, Inc., 1996, p. 824.

[19] A. Erdemir, M. L. Viveiros, J. S. Ulbrecht, and P. R. Cavanagh, “An inverse finite-element model of heel-pad indentation,” *Journal of Biomechanics*, vol. 39, no. 7, pp. 1279-1286, 2006.

Licensing

Copyright (c) 2010 Open Knee Development Team

Unless noted otherwise, this document and the accompanying source code and binary files are licensed under the Creative Commons Attribution-Share Alike 3.0 United States License. To view a copy of this license, visit http://creativecommons.org/licenses/by-sa/3.0/us/; or, (b) send a letter to Creative Commons, 171 2nd Street, Suite 300, San Francisco, California, 94105, USA.

Trademarks and Ownerships

The trademarks (registered or not) listed in this document are the property of their respective owners and are protected by national and international laws on intellectual property and trademark. Abaqus is by SIMULIA (Providence, RI, USA), a Dassault Systèmes brand. Orthone is by ONI Medical Systems, Inc. (Wilmington, MA, USA). Rhinoceros is by McNeel North America (Seattle, WA, USA). TrueGrid is by XYZ Scientific Applications, Inc. (Livermore, CA, USA). FEBio, PreView, and PostView are by Musculoskeletal Research Laboratories, University of Utah (Salt Lake City, UT, USA). SALOME is by Open CASCADE (Guyancourt, France). VolSuite is by Jason Bryan for the Ohio Supercomputing Center (Columbus, OH, USA) and the Cleveland Clinic (Cleveland, OH, USA). Python is by Python Software Foundation (Wolfeboro Falls, NH, USA). SciPy is sponsored by Enthought, Inc. (Austin, TX, USA). Python(x,y) is by Pierre Raybaut. SimTK and Simbios are by the National NIH Center for Biomedical Computing at Stanford (Palo Alto, CA, USA). AMD and Opteron are by Advanced Micro Devices, Inc. (Sunnyvale, CA, USA). Linux is a trademark registered to Linus Torvald.
